# Multicellular simulations with shape and volume constraints using optimal transport

**DOI:** 10.1126/sciadv.adv2781

**Published:** 2026-07-10

**Authors:** Antoine Diez, Jean Feydy

**Affiliations:** ^1^ RIKEN Center for Interdisciplinary Theoretical and Mathematical Sciences (iTHEMS), RIKEN iTHEMS, Wako, Saitama 351-0198, Japan.; ^2^ Institute for the Advanced Study of Human Biology (ASHBi), Kyoto University Institute for Advanced Study, Kyoto University, Yoshida-Konoe-cho, Sakyo-ku, Kyoto 606-8501, Japan.; ^3^ HeKA team, Inria Paris, Inserm, Université Paris-Cité, PariSanté Campus, 2-10 rue d’Oradour-sur-Glane, 75015 Paris, France.

## Abstract

Many living and physical systems such as cell aggregates, tissues, or bacterial colonies behave as unconventional systems of particles that are strongly constrained by volume exclusion and shape interactions. Understanding how these constraints lead to macroscopic self-organized structures is a fundamental question in, e.g., developmental biology. Here, we introduce a framework to model particle systems with arbitrary volumes, dynamical shapes, and deformability properties. Our method is grounded in optimal transport theory and its recent applications in incompressible fluid flows, crowd dynamics, and material sciences. Our approach supports a wide range of interaction and deformation mechanisms, while automatically taking care of the volume exclusion constraint with state-of-the-art numerical performance. We showcase the versatility of this approach through a series of experiments, demonstrating how it extends and refines results from previous approaches, with a special focus on challenging 3D situations in biophysics.

## Teaser

Optimal transport provides a fast and versatile mathematical approach for biophysics simulations of multicellular systems.

## INTRODUCTION

From the smallest intracellular scale (*1*) to the macroscopic population scale (*2*), shape, deformations, and congestion effects play a central role in the dynamics of living systems. For example, cells, bacteria, and other microorganisms appear in a wide variety of shapes and may undergo large deformations, leading to complex spatial organization (*3*, *4*) and collective motion (*5*). Understanding and measuring these effects is a challenging but important experimental problem, especially in developmental biology (*6*, *7*). Over the past decades, in complement to in vitro and in vivo experimental studies (*8*), these questions have been studied using mathematical modeling. In silico experiments let us assess the impact of conjectured biophysical laws in minimal and cheap experimental settings where the shape and physical properties of each agent can be controlled individually. This poses a modeling and implementation challenge as that there is no unique nor simple mathematical way of representing dynamical shapes and multicellular systems. Depending on the objective, it requires to find an appropriate trade-off between the level of details to achieve sufficient physical accuracy, the numerical cost, and the mathematical analytical potential.

We propose a mesoscale approach to simulate efficiently systems of tens to tens of thousands of soft-bodies. This roughly corresponds to the appropriate scale to study collective effects such as swarming or tissue organization during development. Before explaining the details, we briefly review other common models and refer to (*9*–*11*) and to the introductions of (*12*, *13*) for an in-depth discussion of these approaches.

### Point-particle systems

Point-particle systems only consider the dynamics of spatial coordinates and finite-dimensional shape descriptors such as polarity vectors, aspect ratio, etc. This formal simplicity is mathematically and numerically appealing but may not be fully realistic. They are typically more appropriate for very large systems at the statistical physics scale (*5*, *14*).

### Phase fields

Phase fields models can represent arbitrary shapes as an indicator function, which satisfies an Allen-Cahn equation (*15*, *16*). Strongly connected approaches consider the evolution of the boundary of a shape, defined as a curve (*17*–*19*) or as the level-set of a potential function (*12*, *20*–*22*). Contact interactions may be numerically and mathematically challenging and these approaches are usually most efficient for a small number of individuals.

### Cellular automata and lattice models

Cellular automata and lattice models as introduced by Glazier and Graner (*23*, *24*), consider a discretized spatial domain where each “voxel” represents either one biological cell or a portion of it. The voxel allocation and flipping dynamics are defined using custom energy minimization rules.

### Voronoi tessellation

Voronoi tessellation is an off-lattice tiling of the space by polygonal shapes, which has been introduced in biology in (*25*, *26*). Although often realistic, recent experimental observations (*27*, *28*) have motivated the development of refined dynamical models (*29*–*31*). Our approach generalizes this line of work to flexible, nonpolygonal shapes.

### Vertex models

Vertex models define a tiling of the space via a set of vertices connected by straight edges in two dimensions (2D) or plane surfaces in 3D (*32*). The motion of each vertex typically results from an energy minimization hypothesis. More generally, defining an arbitrarily fine meshing of, e.g., the cell membrane, the cell interior, and/or the cell environment allows a particularly accurate physical description, but potentially at a higher numerical cost and mathematical complexity, in particular to treat topological changes. Such approaches are often referred as a finite element method (*33*–*35*) or deformable cell models (DCMs) (*36*–*38*). For the applications, most of these methods are available as open source softwares (*34*, *36*–*45*).

### Our approach

Be it for cells in multicellular aggregates, microorganisms, or pedestrians, the first measurable quantity is the volume. As a starting point, our method revolves around an independent volume constraint for each cell. Most of the approaches described above rather preserve cell volumes using soft constraints and relaxation forces towards a preferred size. Voronoi tessellation methods do not usually consider a volume constraint, or only provide little control on this quantity (*29*). As a first description, our method can be seen as a generalized Voronoi tessellation method with strict volume constraints. To achieve this goal, we rely on the notion of Laguerre tessellation, which has recently appeared in various different contexts, in particular the simulation of incompressible fluid flows (*46*–*48*), of crowd motion (*2*, *49*), and the modeling of polycrystalline materials (*50*–*52*). We also mention impressive applications in computer graphics (*53*–*55*), where a particular case of Laguerre tessellations is known as power diagrams.

Although apparently fundamentally different, these situations are actually related to the theory of optimal transport. Originally developed for operations research and economics by Monge and Kantorovich, this theory describes the most effective way of allocating resources (canonically, piles of sand, or flour) from one location to another while minimizing a transportation cost that is a function of the distance (*56*–*58*). The first connection between optimal transport and fluid mechanics is due to Brenier (*46*). In the modern implementation of Brenier’s ideas (*47*, *48*, *59*), the notion of Laguerre tessellation appears as the natural spatial discretization procedure that preserves the core incompressibility constraint. Similar ideas are used in (*2*, *49*) to model crowd motion, where volume exclusion is crucial. More recently, and as a direct inspiration for our work, Laguerre tessellations and optimal transport have been applied to materials science (*50*–*52*, *60*), where, similarly to biological cell aggregates, some materials like steel are made up of a collection of tiny “crystals” with various shapes and sizes. On the implementation side, we leverage the divide-and-conquer “multiscale” strategy (*56*, *61*, *62*) commonly adopted in optimal transport. Our implementation relies on massively parallel graphics processing units (GPUs), with a state-of-the-art solver accessible through a convenient Python interface (*63*).

**Fig. 1.**
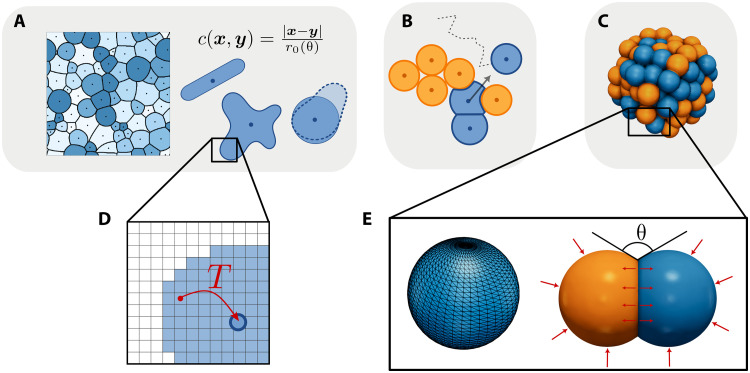
Graphical abstract. (**A**) Laguerre tesselations generalize Voronoi diagrams and level-set approaches with volume, shape, and deformation constraints encoded in a cost function *c*, which can be customized and dynamic. (**B**) Any active point-particle model can be implemented with additional arbitrary softness and deformation properties. (**C**) The framework is independent of the dimension and is implemented in 3D. (**D**) Laguerre tessellations are computed as the solution of a semi-discrete optimal transport problem on a discrete grid, resulting in a map *T*, which assigns each voxel to a cell. (**E**) In 3D, a meshing of each cell boundary is computed to implement surface tension effects and cell sorting mechanisms.

Although our model is formally a tessellation model, we show that it shares important properties with point-particle systems, level-set methods, and vertex models, which thus also suggests a optimal transport point of view for these methods. As a consequence, our model is remarkably versatile: Within the same framework, we can represent individual particles with arbitrary shapes (as in level-set methods), their collective motion (as in point-particle systems), and much denser tissue-like aggregates whose dynamics is ruled by surface tension and other contact-based interactions (typically treated with DCMs). The graphical abstract Fig. 1 summarizes our approach.

## RESULTS

### Static model: Laguerre tessellation

We consider a set of i=1,…,N particles, each of them defined by the couple $\left(x_{i},v_{i}\right)$ of its position, in a given domain Ω, and its volume. By convention, we assume that the total volume of the domain is normalized to 1. The total volume occupied by the particles should thus be $V=v_{1}+\ldots +v_{N}\leq 1$. The main idea of the present article is to model the space occupied by each particle *i* as a Laguerre cell $L_{i}$, defined as the set of points which satisfy the following set of inequalities

$$L_{i}=\left\{x\in \Omega ,\;c\left(x,x_{i}\right)-w_{i}\leq c\left(x,x_{j}\right)-w_{j}\;\text{for}\;\text{all}\;j\right\}$$ (1)

1) The function c:Ω×Ω→[0,+∞) is an arbitrary function, called cost function, which will encode both the shape and the deformability properties of each individual particle. One can instead consider *N* functions $c_{i}:\Omega \to \left[0,+\infty \right)$ but we will mostly consider the case $c_{i}\left(x\right)=c\left(x,x_{i}\right)$ here. For the time being, a typical example to keep in mind is the *L*^2^ cost defined as the square of the distance function: $c\left(x,y\right)={|x-y|}^{2}$.

2) The Kantorovich potentials $w_{1},\ldots ,w_{N}$ are uniquely defined to satisfy the volume constraint $|L_{i}|=v_{i}$.

For a large class of cost functions and any positions **x***_i_*, the Laguerre tessellation (Eq. 1) can be shown to be the unique solution of the following constrained minimization problem on the set of partitions of Ω

$$T_{c}=\underset{{\left(L_{i}\right)}_{i=1,\ldots ,N}}{\text{min}}\left\{\sum_{i=1}^{N}\int_{L_{i}}c\left(x,x_{i}\right)dx,\;\text{with}\;\text{constraints}\;|L_{i}|=v_{i}\right\}$$ (2)

In optimal transport theory, such partition is then understood as an assignment problem, where each point x∈Ω is assigned to one of the **x***_i_* at a cost $c\left(x,x_{i}\right)$ (see the Supplementary Materials for more details). Using this interpretation, one of our main contributions is a fast numerical method to compute the Kantorovich potentials *w_i_*.

When *c* is the *L*^2^ cost and *w_i_* = 0, we recover the standard definition of a Voronoi diagram. Our Laguerre model can thus be understood as a generalized Voronoi tessellation with strict volume constraints. Generalizing the distance function into an arbitrary function *c* is a key modeling idea which allows arbitrary boundary shapes between neighboring particles, since they are defined by algebraic equations of the form

$$L_{i}\cap L_{j}\subset \left\{c\left(x,x_{i}\right)-w_{i}=c\left(x,x_{j}\right)-w_{j}\right\}$$

**Fig. 2.**
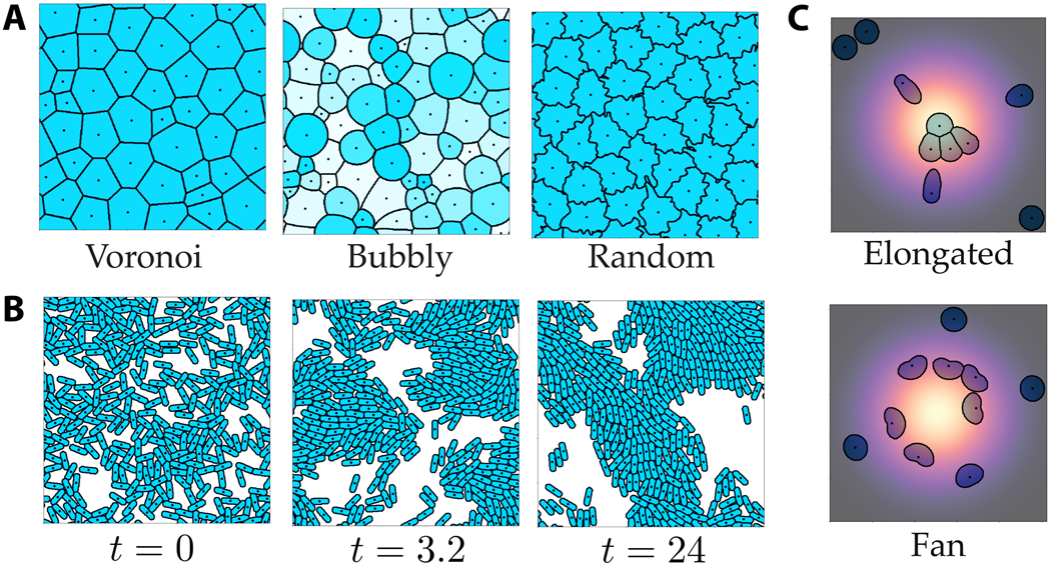
Static and dynamic shapes in 2D. (**A**) Three Laguerre tessellations: (left) Voronoi diagram obtained with the *L*^2^ cost and random volumes *v_i_* sampled uniformly with a ratio 1/5. (Center) A bubbly tessellation similar to (*80*) obtained with 66 particles with random volumes sampled uniformly with a ratio 1/20 and power costs (Eq. 8) with exponents distributed uniformly between 0.5 and 4. Lighter colors indicate lower values of this exponent and correspond to softer shapes. (Right) Voronoi tessellation with random fluctuations similar to (*28*) obtained with 42 identical particles and a randomly perturbed *L*^2^ cost. (**B**) A swarm of rod-shaped particles obtained with the cost (Eq. 7) showing the emergence of long-range alignment due to deformations and nonoverlapping interactions. See also movie S5. (**C**) Chemotaxis motion induced by shape deformations with two choices of biased costs (Eqs. 11 and 12). See also movies S9 and S10.

With the *L*^2^ cost, we recover polygonal shapes, but other choices may lead to more curved boundaries typically observed in biology (Fig. 2A). In the case *V* < 1, we model the empty space as one additional particle **x**_0_ (at an arbitrary location) with volume 1 − *V* and associated to the zero cost *c*_0_(**x**) = 0. The shape (or boundary) of an isolated particle thus corresponds to a level set of the cost function

$$\partial L_{i}=\left\{x\in \Omega ,\;c\left(x,x_{i}\right)=w_{i}-w_{0}\right\}$$

The *L*^2^ cost corresponds to spherical shapes but any shape can actually be realized as the level set of a custom function *c* (Fig. 2, B and C). We will show later that the cost function also encodes how easily these shapes can deform due to collisions in a dynamical framework. This common framework thus connects the so-called generalized Voronoi tessellation models and level-set methods. The optimal transport theory provides a natural way to treat volume constraints, which are otherwise enforced in a soft manner or only heuristically.

### Dynamical model

In a dynamical framework, we first consider the following first-order gradient descent equation for the particles’ locations

$${\dot{x}}_{i}=-\tau_{i}\nabla_{x_{i}}T_{c}=-\tau_{i}\int_{L_{i}}\nabla_{x_{i}}c\left(x,x_{i}\right)dx$$ (3)

where $\tau_{i}\geq 0$ is an arbitrary gradient step and the last equality is proved in (*64*). In fluid mechanics (*46*–*48*), this is the analog of an incompressibility force exerted on each microscopic fluid element. In the present context, this motion leads to repulsion interactions between the particles (movie S1) since the absolute minimum of the total cost $T_{c}$ (Eq. 2) is achieved when the particles are all isolated (if space allows). For the *L*^2^ cost, the incompressibility force points in the direction of the barycenter of the Laguerre cell. Unlike point-particle models, this force is not a sum of binary forces between two locations: It takes into account the actual shapes and contact surfaces between all the particles.

If the cost function only depends on the connecting vector (which will always be the case), i.e., $c\left(x,x_{i}\right)\equiv c\left(x_{i}-x\right)$, then $\nabla_{x_{i}}c\left(x,x_{i}\right)=-\nabla_{x}c\left(x,x_{i}\right)$ and Stokes’ theorem gives an alternative expression for the incompressibility force as an integral over the boundary $\partial L_{i}$ of $L_{i}$

$$-\nabla_{x_{i}}T_{c}=-\int_{\partial L_{i}}c\left(x,x_{i}\right)\vec{n}\mathrm{d\sigma}\left(x\right)$$

where $\vec{n}$ denotes the inward normal and σ the surface measure. This expression shows that the incompressibility force can also be interpreted physically as a resulting internal pressure force, where the pressure on each surface element is proportional to the cost function. Large deformations are thus naturally penalized when the cost is a nondecreasing function of the distance. It also implies that the equations of motion (Eq. 3) preserve the total momentum in the absence of active forces, boundary and when $\tau_{i}\equiv 1$. Summing over *i* and denoting by $\Gamma_{\mathrm{ij}}=L_{i}\cap L_{j}$ the boundary between two Laguerre cells,

$$\sum_{i=1}^{N}{\dot{x}}_{i}=\sum_{\left\{i,j\right\}}\int_{\Gamma_{\mathrm{ij}}}\left\{c\left(x,x_{j}\right)-c\left(x,x_{i}\right)\right\}\;\vec{n}\text{dσ}$$

where the sum is over all unordered pairs of indices and $\vec{n}$ points towards the *i*th cell. By definition of the Laguerre cells, the integrand is constant on Γ*_ij_*, equal to *w_j_* − *w_i_* so that

$$\sum_{i=1}^{N}{\dot{x}}_{i}=\sum_{i=1}^{N}w_{i}\int_{\partial L_{i}}\vec{n}\mathrm{d\sigma}=0$$

since $\partial L_{i}$ is a closed surface.

We can take advantage of this clear mathematical structure to incorporate any other force or noise terms. We will consider general first-order (stochastic) differential equations systems of the form

$${\dot{x}}_{i}=-\tau_{i}\nabla_{x_{i}}T_{c}+F_{i}^{\text{point}}\left(x_{1},\ldots ,x_{N}\right)+F_{i}^{\text{surf}}\left(L_{1},\ldots ,L_{N}\right)+{\dot{\xi }}_{i}$$ (4)

The force term $F_{i}^{\text{point}}$ includes all the external or interaction forces which depend only on the locations, as in point-particle models: e.g., gravity, self-propulsion, attraction, etc. The force term $F_{i}^{\text{surf}}$ on the contrary may depend on the shapes of the particles and can typically model pressure forces and surface tension effects along each interface. Stochastic effects can be included in the force terms or by adding a stochastic noise ξ*_i_*. Note that for any dynamics, the nonoverlapping and volume constraints are automatically ensured at all times by the definition of the Laguerre cells (Eq. 1).

One can also consider dynamical volumes *v_i_* or cost functions (i.e., shapes) *c_i_*. Just as for the position **x***_i_*, the time derivative of the volume ${\dot{v}}_{i}$ may depend on all the components of the model thus allowing arbitrary growth behaviors. For the dynamics of the cost, we introduce two (nonexclusive) main modeling ideas.

1) Parametrized costs: $c_{i}\left(x\right)=c\left(x,x_{i};P_{i}\right)$ where $P_{i}$ is an arbitrary set of parameters: typically they will model an orientation. A prototypical example is

$$c\left(x,x_{i};\Sigma_{i}\right)={\left(x-x_{i}\right)}^{T}\Sigma_{i}^{-1}\left(x-x_{i}\right)$$ (5)

where Σ*_i_* is a covariance matrix, which in this case imposes an ellipsoid shape. The parameters $P_{i}$ can have an arbitrary dynamics, as in classical active particle models, but can also possibly depend on the shapes $L_{i}$.

2) Level-set potential functions: $c_{i}=e^{-\varphi_{i}}$ where $\varphi_{i}=\varphi_{i}^{0}+f_{i}$ is a potential functions, which is a modification of a base potential $\varphi_{i}^{0}$ with a perturbation *f_i_*. While $\varphi_{i}^{0}$ defines the base shape of the particle, the perturbation *f_i_* introduces a deformation bias in arbitrary (potentially time-varying) directions, typically given by external clues such as a chemotactic field.

Note that the optimization step ensures the strict volume constraint thanks to the unique choice of the potentials *w_i_* in Eq. 1, while the incompressibility force (Eq. 3) tends to restore the preferred shapes. The approach thus differs from Voronoi-based models (*30*) where the potentials *w_i_* = 0 are always kept constant and the incompressibility constraint is instead enforced using an additional energy minimization principle. However, in both models, the main parameters are the centroid positions **x***_i_* that follow equations of motion of the form (Eq. 4). Finally, let us mention that since the cell shapes are instantaneously computed as a Laguerre cell (i.e., the relaxation of cell shapes occurs within a single simulation step), the framework implicitly assumes that the relaxation of the interface shape is much faster than the relaxation of the cell-center positions **x***_i_*. Under this approximation, this model does not describe the intrinsic relaxation dynamics of the interface shape itself.

In the following, we provide several examples to showcase the applicability and versatility of our model in comparison to other computational models. All these examples are based on Eq. 4, The detailed numerical values of the parameters and implementation details regarding the computation of the cost, of the interaction forces, and of the boundary conditions are gathered in Materials and Methods.

### Soft-body simulations: First examples and benchmark

**Fig. 3.**
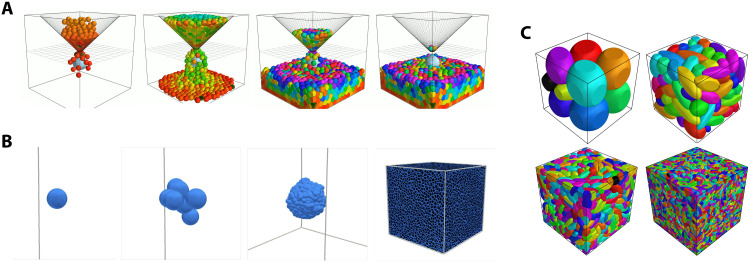
3D benchmarking examples. (**A**) Falling soft-spheres in an hourglass domain. See also movie S2. (**B**) Exponential growth of a 3D aggregate via successive cell division and growth phases, zooming out from *N* = 1 to *N* = 50, 000 cells. See also movie S3. (**C**) Final configuration of a deformation-driven run-and-tumble motion for *N* = 10, 100, 1000, and 10,000 deformable ellipsoids with a space discretization grid of size *M* = 512. See also movie S4.

As a first illustration, we start with the *L*^2^ cost and consider a simple free-fall motion corresponding to $F_{i}^{\text{point}}=-e_{z}$ and $F_{i}^{\text{surf}}=0$. To emphasize the deformations, we consider an hourglass domain Ω. In this active Voronoi model, particles tend to keep spherical shapes and their contact surfaces are planes (Fig. 3A).

As a classical test case in computational biology (*4*, *13*, *15*, *36*, *65*), we consider the growth of a cellular aggregate, with no external forces and only the volume exclusion repulsion force modeled by Eq. 3. Initially a single cell grows linearly. When it reaches a target volume and after a random exponential time, this cell divides along a random division plane in two daughter cells with equal half volumes and which follow the same dynamics. The volume exclusion interactions lead to an exponentially growing spherical aggregate (Fig. 3B). We end the simulation when its volume entirely fills a given box domain, reaching *N* = 50,000 particles. Using our implementation, the total computation time is about 1 day.

As a more detailed benchmarking experiment, we consider a system of self-propelled 3D deformable ellipsoids constrained in a box domain (Fig. 3C). The orientation of each particle is given by its covariance matrix Σ*_i_*, which defines both its direction of motion and aspect ratio. The ellipsoidal shape is enforced by the parametrized cost (Eq. 5). We consider a strongly packed situation: The covariance matrix of each particle is defined at each time using the principal components analysis (PCA) of its current Laguerre cell. This results in a run-and-tumble motion (movie S4). Table 1 shows the simulation time for 2000 iterations depending on the number of particles *N* and the space discretization grid *M* (see Materials and Methods). The complexity is linear in *N* and *M^d^* but the GPU implementation is suboptimal for small *N*.

**Table 1.** Benchmark. Total computation time for 2000 iterations and various values of *N* and *M* in dimension *d* = 3. h, hour.

	*M*			
*N*	64	128	256	512
10	30 s	1.3 min	7.4 min	62 min
100	45 s	1 min	4.7 min	36 min
1,000	NR	4 min	25 min	3 h
10,000	NR	NR	4.9 h	35 h

### Emergence of orientational order for rod-shape active particles

Oriented particles with anisotropic shapes can be realized in our optimal transport framework by considering appropriate cost functions. In dimension 2, any shape defined by a polar equation $r=r_{0}\left(\theta \right)$ can be encoded by the cost

$$c\left(x,x_{i}\right)={\left\{\frac{|x-x_{i}|}{r_{0}\left[\theta \left(x,x_{i}\right)\right]}\right\}}^{\alpha }$$ (6)

where $\left(x,x_{i}\right)$ denotes the polar angle of the vector **x** − **x***_i_* and α > 0 is a hardness parameter whose influence will be illustrated later. The extension to dimension 3 is straightforward. An important example is the spherocylinder shape of 2D bacilli and rod-shape polymers. Given a long-axis vector ${\left(\text{cos}\;\theta_{0},\text{sin}\;\theta_{0}\right)}^{T}$ and an aspect ratio s=a/b≥1, this shape can be realized using the cost associated to the function

$$\begin{array}{c} r_{0}\left(\theta \right)=\\ \left\{\begin{array}{c} \frac{b}{|\text{sin}\left(\theta -\theta_{0}\right)|},\;\text{if}\;|\text{tan}\left(\theta -\theta_{0}\right)|\geq \frac{1}{s-1},\\ b\left\{\left|\text{cos}\left(\theta -\theta_{0}\right)\right|\left(s-1\right)+\sqrt{1-{\left[\left(s-1\right)\text{sin}\left(\theta -\theta_{0}\right)\right]}^{2}}\right\},\text{otherwise} \end{array}\right. \end{array}$$ (7)

Using Eq. 6, we model a system of self-propelled swarming bacteria. The cost is parametrized by the long-axis vector which also defines the direction of motion. It is defined at each time as the leading vector of the PCA of the current Laguerre tessellation. This motion corresponds to “bending” and turning effects due to the collisions. With this simple rule, the emergence of orientational order (or alignment) is observed from the sole volume exclusion interactions, as classically hypothesized in polymer physics (Fig. 2B with *N* = 300 and fig. S7C with *N* = 150). A similar idea has recently been developed in (*66*) in a computational model of deformable ellipsoidal particles representing fibroblasts.

This emergent order can classically be evaluated quantitatively by computing the time evolution of the nematic order parameter $\phi =\frac{d}{d-1}\lambda \in \left[0,1\right]$ where λ is the largest eigenvalue of the matrix obtained by averaging the individual *Q*-tensor matrices $Q_{i}=n_{i}⊗n_{i}-\frac{1}{d}\text{Id}$, with the dimension *d* = 2 and **n***_i_* the long-axis vector of particle *i*. For the larger system (Fig. 2B), a sharp transition is observed around time *t* = 12 and orientational order is preserved until *t* = 25 (fig. S7A). However, for this set of parameters, we have observed that the orientational order is typically not persistent over extended intervals of time but rather fluctuates following the successive formation and disappearance of ordered clusters (movie S5). A persistent orientational order can be observed in the smaller (*N* = 150) but denser system (see fig. S7, B and C, for a simulation of such system until *t* = 600).

### Fluid-solid phase transition

Here, we consider soft spherical particles of equal size associated to power costs of the form

$$c_{\alpha }\left(x,y\right)={\lambda_{\alpha }}^{-1}{|x-y|}^{\alpha }$$ (8)

where λ_α_ > 0 is a normalizing factor (see Methods). Here the exponent α plays the role of a deformability parameter. Indeed when α > 0 is large, the cost $c\left(x,x_{i}\right)$ of assigning a point **x** to the cell *i* is close to zero for points near **x***_i_*, but it increases sharply for points farther away. On the contrary, when α→0, the cost function becomes flatter and thus assigns a similar cost to any point. Hence a small α allows large deformations while a larger α penalizes deformations.

**Fig. 4.**
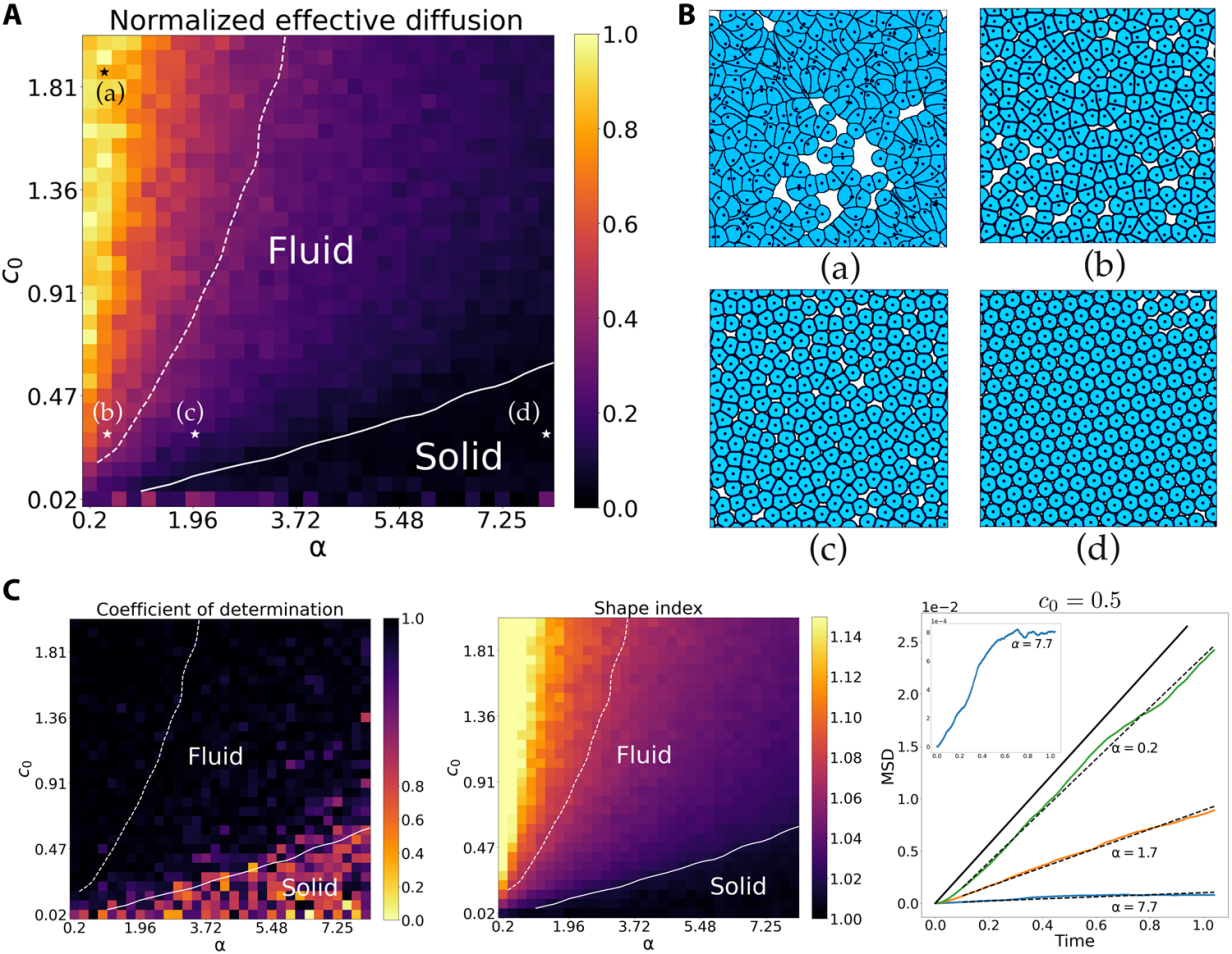
Active Brownian particles with deformations. (**A**) Phase diagram in the $\left(\alpha ,c_{0}\right)$ plane. (**B**) Snapshots of the final configuration for different values of $\left(\alpha ,c_{0}\right)$ indicated by the labels (a), (b), (c), and (d) on the phase diagram. See also movies S6 to S8. (**C**) (left) Coefficient of determination of the linear fit in the $\left(\alpha ,c_{0}\right)$ plane. The center of the color map is set at *r*^2^ = 0.9. (center) Average shape index $\langle \sigma_{i}\rangle$ in the $\left(\alpha ,c_{0}\right)$ plane. The center of the colormap is set at $\sigma =3.81/\left(2\sqrt{\pi }\right)$. (Right) MSD (plain line) and linear fit (dashed line) over time for *c*_0_ = 0.5 and α=0.2,1.7,7.7. The black line has slope *D*_thr_. Insert: zoom on the MSD curve α = 7.7 showing saturation when the system has reached a stable hexagonal pattern. See also fig. S8A for the same plot but over a longer time interval.

As in (*17*, *30*), we confirm that the cell deformability parameter α drives a fluid-solid phase transition in a 2D system of active Brownian particles. We simulate the following system with *N* = 250 in the square periodic domain $\Omega ={\left[0,1\right]}^{2}$

$${\dot{x}}_{i}=c_{0}n_{i}-\tau \nabla_{x_{i}}T_{c_{\alpha }},\;{\dot{n}}_{i}=\sqrt{2D}{\dot{\xi }}_{i},\;|n_{i}|=1$$ (9)

where *c*_0_ > 0 is the self-propulsion speed and *D* is the diffusion of the direction of motion. A single point particle (τ = 0) has a purely diffusive behavior with a mean-square displacement (MSD) growing linearly in time at speed $D_{\text{thr}}=2c_{0}^{2}/D$. We compute the effective diffusion coefficient *D*_eff_ of the shape constrained system (τ > 0) as the slope of the best linear curve fitting the MSD of the centroids **x***_i_* over time (in the least squares sense, Fig. 4C) after unwrapping the periodic trajectories to the full domain. A few initial steps of Lloyd algorithm ensure that the particles are initially well-scattered. To avoid boundary effects, we simulate the system until the time 0.5/*c*_0_. A longer simulation time produces similar results (fig. S8A). The phase diagram (Fig. 4A) shows the normalized value ${\bar{D}}_{\text{eff}}=D_{\text{eff}}/D_{\text{thr}}$ for small Péclet numbers and constant values *D* = 20 and τ = 3.

The diffusive behavior can be evaluated by the coefficient of determination *r*^2^ of the linear fit (Fig. 4C). The deformation of a Laguerre cell $L_{i}$ is measured as in (*30*) by the shape index $\sigma_{i}=\frac{|\partial L_{i}|}{2\sqrt{\pi }|L_{i}|}\geq 1$ with σ*_i_* = 1 corresponding to a disk (Fig. 4C).

In the fluid phase, the system keeps a diffusive behavior (*r*^2^ > 0.9) despite the volume exclusion interactions but with a lower diffusion coefficient ${\bar{D}}_{\text{eff}}<1$. Note that for *c*_0_ large and α small, the shape constraint is very weak which produces unrealistic nonconnected or engulfed shapes while ${\bar{D}}_{\text{eff}}$ converges to 1 (Fig. 4B). In the solid phase the effective diffusion vanishes and the system reaches a stable hexagonal hard-sphere packing final configuration with an average shape index $\langle \sigma_{i}\rangle$ close to 1. The transition between the fluid and the solid phases (Fig. 4A, white plain line) is computed from the shape index diagram (Fig. 4C) as the level set σ*_i_* = 1.015 where the Laguerre cells become indistinguishable from perfect disks at the resolution considered. In the solid phase, the MSD saturates and the *r*^2^ value of the linear fit drops (Fig. 4C). In addition, a log-log plot of the MSD that shows that the anomalous diffusion exponent remains close to 1 for most values of α is shown in fig. S8B.

The dashed white line corresponds to the level-set of an average shape index $\langle \sigma \rangle =\frac{3.81}{2\sqrt{\pi }}$, which is advocated in (*30*) as a transition point between a fluid and a soft-fluid phase. In our model, as in the contour model (*17*), this valued does not seem to correspond to a sharp transition of the effective diffusion. In (*17*), this special value is rather associated to the percolation of topological defects but as it is not the main goal of the present article, we leave this point for later work.

Such model can be used to simulate crowds and jamming phenomena due to volume exclusion as well as sorting between heterogeneous populations of more or less soft and heavy particles. Such simulations are presented in the Supplementary Material. Moreover, unlike other computational approaches, our model is particularly well-suited for coarse-grained mathematical analysis. On the basis of theoretical results in optimal transport theory, a formal derivation presented in the Supplementary Text leads to a mean-field approximation of the particle system (*9*). It takes the form of a system of partial differential equations that describes the evolution of the density of particles f(x,n) at a position *x* and with the orientation **n**

$$\partial_{t}f\left(x,n\right)+c_{0}\nabla_{x}f=\tau \nabla_{x}\cdot \left(\nabla_{x}\Phi f\right)+D\Delta_{n}f$$ (10A)

$$\text{det}\left(\text{Id}-\nabla_{x}^{2}\ell^{*}\left(\nabla_{x}\Phi \right)\nabla_{x}^{2}\Phi \right)=f$$ (10B)

The potential Φ(**x**) is the solution of a nonlinear Monge-Ampère equation, which is known in other contexts in physics (*67*, *68*). The function $\ell^{*}$ is the Legendre transform of the function $\ell \left(x\right)={|x|}^{\alpha }$.

### Deformation-driven motion

In the level-set formalism (*22*) the boundary of each individual particle is defined as the level-set of a potential function φ, usually initially chosen as the signed distance from the boundary. In our framework, this representation is equivalent to taking a cost of the form $c=e^{-\varphi }$ (since the cost is an intrinsically nonnegative multiplicative quantity). Level-set methods are particularly adapted to model the motion of biological cells induced by the deformation of their membrane, driven for instance by an external chemotactic field. Here, as in (*22*), our goal is not a full description of how this field drives the internal reorganization of the cell’s actin cortex but we rather aim at a minimal phenomenological model able to reproduce different scenarios of cell motion.

We consider a chemo-attractant density *u*(**x**) in Ω and we classically assume that a particle located at **x***_i_* can sense the local gradient $u\left(x,x_{i}\right)$ along the directions **x** − **x***_i_*, defined through a finite difference formula. To model the deformations induced by this field, the dynamics of φ could typically be defined by an advection equation modeling the forces acting on the boundary. However, here, we consider a simpler approach where a deformation term is added to the potential defining a spherical *L*^2^ Laguerre cell

$$\delta u\left(x,x_{i}\right)=\frac{u\left(x\right)-u\left(x_{i}\right)}{|x-x_{i}|},\;\varphi \left(x,x_{i}\right)=-2\text{log}|x-x_{i}|+\beta f\left[\delta u\left(x,x_{i}\right)\right]$$ (11)

for some constant β > 0 and some function *f*, which models how the gradient affects the deformation. This deformation can be orthogonal to or along the chemo-attractant gradient (*22*). Denoting by $\delta_{+}=\text{max}\left(0,\delta \right)$ and $\delta_{-}=\text{max}\left(0,-\delta \right)$ the positive and negative parts of a number δ, two choices for *f* are thus

$$f\left(\delta \right)=-\delta_{+},\;f\left(\delta \right)=\delta_{-}^{2}+\delta_{+}$$ (12)

The first *f* lowers the potential in the directions of large nonnegative gradients. This produces elongated shapes (Fig. 2C and movie S9). The second *f* increases the cost in the direction of both positive and negative gradients but more in the latter case. This produces fan-like shapes (Fig. 2C and movie S10). In both situations, we consider the simplest possible dynamics given by Eq. 3. Since the morphological changes are biased in the direction of increasing gradient, this choice induces chemotactic migration, with the two different cell shape deformations. Both situations are comparable to the ones obtained with the level-set method in (*22*) and are in agreement with the experimental observations therein.

An important point to note is that not only the volume is preserved regardless of the deformation but our model also includes nonoverlapping and cell-cell repulsion. The morphological changes resulting from these mechanical interactions would typically be difficult to model in the level-set formalism, where mostly a single cell or noninteracting cells are considered. They are inherently included in our modeling framework.

### Cell sorting via surface interactions

So far, Laguerre cells have been used to model soft bodies with internal pressure forces that maintain a preferred shape, with a response to deformations that can typically be controlled using the hardness parameter α. In some contexts such as biological cells and bubbles, it is important to also model adhesion phenomena that typically depend on surface tension effects. To do so, another set of forces and parameters need to be introduced. In this section, we thus model large aggregates of deformable spheres using Laguerre cells defined by a simple weighted *L*^2^ cost but interacting via the following forces computed along each interface

$$c\left(x,x_{i}\right)=\frac{\gamma_{i0}}{R_{i}}{|x-x_{i}|}^{2},\;F_{i\leftarrow j}=\int_{\Gamma_{\mathrm{ij}}}\left(\gamma_{\mathrm{ij}}|\kappa |+\frac{\eta_{\mathrm{ij}}}{|x_{i}-x_{j}|}\right)\vec{n}\;\text{dσ}$$ (13)

The force $F_{i\leftarrow j}$ results from elementary pressure-like interactions depending on the local mean curvature κ along each interface $\Gamma_{\mathrm{ij}}=L_{i}\cap L_{j}$ between the Laguerre cells *i* and *j* (with possibly *j* = 0). The vector $\vec{n}$ denotes the inward normal of $L_{i}$ so this force is always a repulsion force. By moving the centroid **x***_i_* away from its interface, the first term reduces the local curvature while the second reduces the interface area. The parameters $\gamma_{\mathrm{ij}},\eta_{\mathrm{ij}}>0$ have the dimension of surface tensions. When *j* = 0 (interaction with the medium), we set $\eta_{i0}=0$ so that only the curvature term remains. The surface tension γ_*i*0_ between a cell and the medium is also assumed to control the relative softness between the particles by defining the weight of the cost function. The cost is nondimensionalized by dividing by the radius *R_i_* of the particle *i*. Larger particles with a smaller surface tension parameter thus appear relatively softer. The choice of the *L*^2^ cost ensures that each interface has a constant curvature. The total force exerted on **x***_i_* is finally defined as

$$F_{i}^{\text{surf}}=\sum_{j=0}^{N}F_{i\leftarrow j}-\frac{1}{N_{i}}\sum_{k\in N_{i}}F_{k\leftarrow 0}$$

where $N_{i}$ is the connected aggregate to which *i* belongs and *N_i_* denotes the number of cells in $N_{i}$. The second term on the right-hand side is a correction term. Although for two Laguerre cells, $F_{i\leftarrow j}=-F_{j\leftarrow i}$, there might be nonzero momentum gain coming from the interaction with the medium so we chose to evenly split it within the cell aggregate to restore the conservation of momentum. Note that this is a consequence of the simplifying approach to treat the medium as one single particle with zero cost. Alternatively, the medium could be treated as an incompressible fluid discretized using a Laguerre tessallation as in (*47*, *48*), which would preserve momentum but would be numerically much more costly.

This model satisfies the Young-Dupré relationship which states that the equilibrium configuration of two identical particles is a cell doublet made of two spherical caps (Fig. 1E) intersecting with a contact angle θ∈[0,π] defined by

$$\text{cos}\frac{\theta }{2}=\frac{\eta }{2\gamma }$$

We further validate this model by reproducing various cell sorting phenomena observed in biology which are commonly used as a standardized test case for computational models (*10*, *12*, *23*, *24*, *26*, *69*, *70*) since the work of Chen and Brodland on the so-called differential interfacial tension hypothesis (*33*, *69*, *71*). While most models in the literature are intrinsically 2D, we showcase the applicability of our approach in a fully 3D setting.

Following Chen and Brodland, we thus consider an initial homogeneously mixed aggregate with two types of cells denoted by *b* and *o* (respectively for blue and orange), embedded in a medium (Fig. 1C). The parameters γ and η are assumed to depend only on the cell type. Our model thus has six parameters denoted by $\gamma_{\mathrm{oo}},\gamma_{\mathrm{bb}},\gamma_{\mathrm{ob}},\eta_{\mathrm{oo}},\eta_{\mathrm{bb}},\;\text{and}\;\eta_{\mathrm{ob}}$ plus two surface tension parameters denoted by γ*_b_* and γ*_o_* between each type and the medium. Since the interface between two cells of the same type has zero curvature, we can set $\gamma_{\mathrm{bb}}=\gamma_{\mathrm{oo}}=0$. To investigate the phase diagram of this model, we first define several dimensionless parameters. First, the compaction parameters respectively for orange and blue aggregates and a virtual orange aggregate in a medium similar to blue particles are defined using Young-Dupré relation as

$$k_{o}=\frac{\eta_{\mathrm{oo}}}{2\gamma_{o}},\;k_{b}=\frac{\eta_{\mathrm{bb}}}{2\gamma_{b}},\;k_{\mathrm{ob}}=\frac{\eta_{\mathrm{oo}}}{2\gamma_{\mathrm{ob}}}$$

Then, the ratio $\bar{\gamma }=\gamma_{o}/\gamma_{b}$ defines the relative softness between the two cell types. Without loss of generality, we assume that $\bar{\gamma }\geq 1$ (i.e., the orange cells are relatively harder). Last, the ratio $\bar{\eta }=\eta_{\mathrm{ob}}/\eta_{\mathrm{oo}}$ defines the relative repulsion strength between orange and blue cells and between orange cells.

**Fig. 5.**
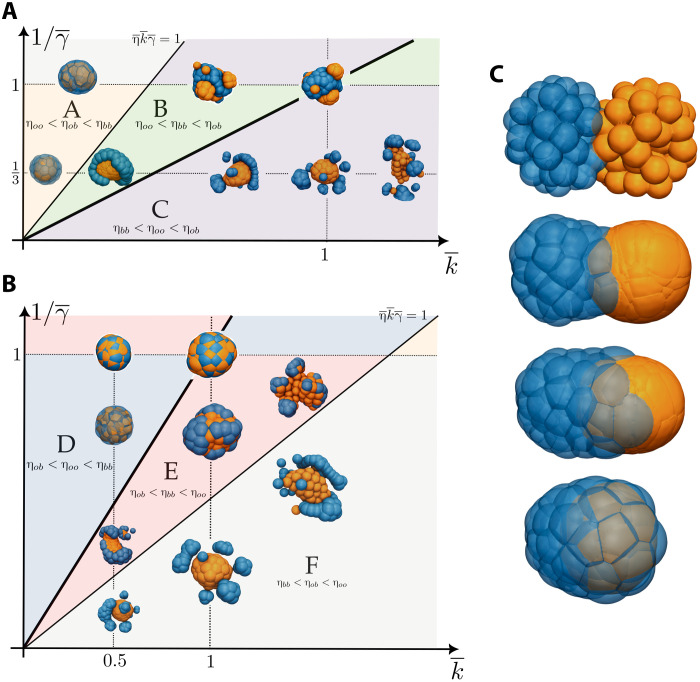
Sorting patterns in 3D. (**A** and **B**) Sorting patterns in a homogeneous mixture of two cell types with resp. η=3>1 and η=0.3<1, obtained from an initial mixed aggregate of *N* = 120 cells, under different conditions on the surface tensions parameters. The phase domain compares relative compactness $\bar{k}$ with relative softness $\bar{\gamma }$. The line $\left\{\bar{k}\bar{\gamma }=1\right\}$ defines the boundary between the regions $\left\{\eta_{\mathrm{oo}}≶\eta_{\mathrm{bb}}\right\}$ and the line $\left\{\bar{k}\bar{\gamma \eta }=1\right\}$ defines the boundary between the regions $\left\{\eta_{\mathrm{ob}}≶\eta_{\mathrm{bb}}\right\}$. Two disconnected regions with the same background color are equivalent with blue and orange cells inverted depending on $\bar{\gamma }≷1$. (**C**) Engulfment of an aggregate of orange cells in an aggregate of blue cells starting from a configuration where the two cell types are separated (region A: $\bar{\eta }=3$, $\bar{\gamma }=2$, and $\bar{k}\bar{\gamma \eta }=0.8$), see also movie S14.

From now on, we take the blue cells as a reference and set $\gamma_{b}=1$ and $k_{b}=k_{\mathrm{ob}}=0.4$. We vary the three remaining ratios $\bar{\eta }$, $\bar{\gamma }$, and $\bar{k}=k_{o}/k_{b}$, which conveniently partitions the phase space into six regions (labeled A to F in Fig. 5, A and B) obtained by permuting the ordering of the repulsion strengths $\eta_{\mathrm{oo}},\eta_{\mathrm{ob}},\;\text{and}\;\eta_{\mathrm{bb}}$. They are depicted in the two phase diagrams in the $\left(\bar{k},{\bar{\gamma }}^{-1}\right)$-plane for $\bar{\eta }>1$ (i.e., the orange cells have a stronger affinity with their own type, Fig. 5A) and $\bar{\eta }<1$ (i.e., the orange cells have a stronger affinity with the other type, Fig. 5B).

The results show that our model can reproduce at least all the configurations described in (*3*, *69*) namely, homophilic sorting with total or partial engulfment of one of the populations (respectively, A and D and B and E), homophilic sorting with separation by the medium (C and F), heterophilic/checkerboard sorting (D and E when $\bar{\gamma }$ tends to 1). More precisely, when $\bar{\eta }>1$, decreasing the compaction of the harder particles (i.e., increasing $\bar{k}$) makes the cell aggregate evolve from total engulfment (A) to partial engulfment (B) and separation (C), until the total dissociation of the aggregate by the medium when $\bar{k}>1$ (Fig. 5A). In particular, in the region A, the orange aggregate is highly compact but the repulsion force between orange and blue cells is relatively lower ($\eta_{\mathrm{ob}}<\eta_{\mathrm{oo}}$), which drives progressive engulfment, even from an initial segregated configuration (Fig. 5C) as in (*12*, *69*). When $\bar{\eta }<1$ transitions are better visualized along the $1/\bar{\gamma }$ axis. For similar cells ($\bar{\gamma }=1$ and $\bar{k}<1/\bar{\eta }$), the heterophilic interactions [$\eta_{\mathrm{ob}}<\text{min}\left(\eta_{\mathrm{oo}},\eta_{\mathrm{bb}}\right)$] produces a checkerboard patterning. Increasing the relative hardness of the orange cells is equivalent in our model to increase the repulsion strength $\eta_{\mathrm{oo}}$ and decrease $\eta_{\mathrm{bb}}$, which eventually leads from an engulfed/checkerboard state (D) to partial engulfment (E) and separation (F). Decreasing the compaction of the orange cells (i.e., increasing $\bar{k}$) prevents engulfment as it could be expected (Fig. 5B). Representative situations are shown in movies S11 to S14.

In the previous example, the cells have equal volume so their interface is always a plane and the interfacial terms with $\gamma_{\mathrm{ob}}$ vanish (curvature κ = 0). In the fig. S9, we show the equilibrium configuration for a pair of Laguerre cells with $\gamma_{\mathrm{ob}}=1\neq 0$ and different values of the volume ratio and compaction parameter $k≔\frac{\eta_{\mathrm{ob}}}{2\gamma_{o}}$. Here, we assume that larger particles are relatively softer (which comes from the definition of the cost function that prescribes the geometry of the cells and may be understood as a consequence of Laplace pressure although it is not explicitly modeled here). As a consequence, the interface with a smaller particle is curved. When *k* is small, the cells have a stronger affinity. When the cells are identical (fig. S9 right column), the cells may eventually merge into one spherical cell following Young-Dupré relation. When the volume ratio decreases (left column), the smaller cell may eventually be internalized. These results are qualitatively consistent with the model in (*36*, *72*). Note that we do not seek a quantitative comparison here as our system is not based on the same energy minimization formulation; moreover, an extended version of Young-Dupré relation to this case would depend on the physical properties and modeling choices for nonidentical particles with different volumes. As an illustrative example with the simple modeling choices adopted here, we show a visually realistic simulation of bubbles in the Supplementary Material (fig. S5).

## DISCUSSION

We have introduced an approach to model, within a same framework, diverse systems ranging from individual deformable cells to complex tissue aggregates. The core idea is the notion of Laguerre tessellation in connection with optimal transport theory. Our model supports arbitrary shapes and physical deformations with a strict volume constraint, allowing complex soft-body simulations, with applications in particular in computational biology. Although our method is formally an extension of the idea of Voronoi tessellation, we have highlighted links with other independent methods such as point-particle systems, level-set methods and DCM. We also provide an efficient GPU implementation, which leverages optimal transport techniques.

Since, to the best of our knowledge, our approach is new in the literature, the main focus of the present article was to validate it by comparison to other classical computational models, thus providing an independent validation of their conclusions. The next important step is to seek a direct biological validation of our method by the confrontation to experimental data: A first step would be to fit Laguerre tessellations to 3D imaging data, as recently done in (*73*) for 2D material science data. Then, our method seems well suited to the in silico modeling of the development of blastocysts. Recent experimental findings (*6*, *70*) have identified several complex phenomena with morphological changes, sorting phenomena and dynamical topological properties via the formation of lumen. In order to understand the regulation processes between these phenomena, we need a mathematical model that can handle both individual cell deformations and their behaviors among dense aggregates. This seems particularly challenging using traditional methods, or at the price of an unreasonable computational cost, especially in a 3D setting. This biological question was the initial motivation for the development of our method and is still an on-going work.

On the theoretical and numerical sides, several open questions and future improvements could be considered: (i) Since our model has an intrinsic point-particle description, a natural question is to extend the active vertex theory developed in (*26*, *30*, *65*) to the case of Laguerre tessellations with volume constraints. In the same vein, it would be desirable to study whether the interaction mechanisms that we have introduced (or new ones) can be recast into a more common energy minimization framework. The main theoretical and computational difficulties for these endeavors are linked to the differentiability properties of the solutions of semi-discrete optimal transport problems.

(ii) A clear advantage of point-particle descriptions is the potential to derive coarse-grained continuum models better suited to mathematical analysis (*74*). For particles with a volume, this is, to the best of ou knowledge, a largely open question, with the notable exception of (*58*, *75*) in the context of crowd evacuation modeling. A preliminary formal analysis (Supplementary Material) suggests a methodology based on mean-field theory that should lead to a novel continuum approach for soft-body systems.

(iii) Optimal transport theory is historically linked to the design of numerical schemes for fluid flows. Thus, it provides a direct way to include fluid-structure interactions between macroscopic cells immersed in a fluid environment, in the mold of (*21*, *48*). In particular, a discretization of the medium using (*47*, *48*) could address the momentum conservation issues that result from the simplification of the medium as a zero-cost Laguerre cell.

(iv) Although our GPU implementation is already quite efficient and operational for most cases, performance improvements could be considered. In particular, complex dynamical costs may remain challenging to handle, especially when they are defined implicitly as in level-set methods (*22*) due to our symbolic computation framework (*63*). Moreover surface interactions currently rely on isocontouring algorithms that would benefit from a fully GPU implementation for very large systems. On the basis of current benchmarking results, these improvements could lead to the development of a real-time 3D simulation tool for computational physics and biology.

## MATERIALS AND METHODS

### Implementation

Our implementation framework is structurally similar to the so-called active vertex models (*20*, *65*), simply looping back and forth between the computation of the space tessellation and the force terms.

Starting from a set of locations, volumes and cost functions, we first compute an initial Laguerre tessellation. A classical procedure to ensure realistic initial configurations is known as Lloyd algorithm (*50*, *64*). Then, the general Eq. 4 is discretized in time using an explicit Euler(-Maruyama) scheme with a small time-step Δ*t*. At each time step, we use the current Laguerre tessellation to compute all the force terms needed to update the locations, volumes, and costs. These parameters are then used to compute the new current Laguerre tessellation.

The most expensive part is the computation at each time step of a new Laguerre tessellation. We use a fast optimal transport solver implemented on the GPU, which relies on a fine space discretization of the domain (Fig. 1D and fig. S1) as explained below. The full code, with an API, a documentation and a gallery of examples can be found on GitHub and at https://iceshot.readthedocs.io/.

### Optimal transport solver

Whether it is in computational fluid dynamics or in materials science, the semi-discrete optimal transport problem is considered mostly for the squared Euclidean cost $c\left(x,y\right)={|x-y|}^{2}$. Extremely efficient numerical methods, based on a damped Newton algorithm, have been developed for this case and are now available as open source softwares (see, for instance, the packages Geogram or Pysdot available on GitHub). Unfortunately, these implementations leverage the structure of the squared Euclidean distance to compute Laguerre cells efficiently and cannot be applied with general cost functions.

In our approach, the freedom in the choice of arbitrary, individualized cost functions *c_i_* is of upmost importance. To unlock the use of Laguerre cells with general shapes and deformability properties, we work in a fully discrete setting with a discrete approximation of the domain $\Omega ={\left[0,1\right]}^{d}$ by a uniform grid of *M* “voxel centers” **y***_j_* (Fig. 1D. Nonsquare domains are simply cropped versions of ${\left[0,1\right]}^{d}$. We choose M≫N so that each particle is effectively discretized with a sufficiently high number of voxels **y***_j_* with volume 1/*M*. Solving discrete optimal transport problems is by now classical owing to numerous applications in data science (*57*). A quite reliable and stable method is the symmetrized and annealed variant of the renowned Sinkhorn algorithm (*56*). However, as we target a tight fit to the volume constraints and since the dynamical setting naturally lets us reuse optimal potential values *w_i_* from one time step to another, we rely instead on a solver that leverages the semi-discrete structure of the problem. The main observation, proved for instance in (*64*, *76*), is that the dual potentials $w={\left(w_{i}\right)}_{i=1,\ldots ,N}$ that appear in the definition (Eq. 1) of the Laguerre cells solve the dual maximization problem

$$w=\underset{{\left(w_{i}\right)}_{i=1,\ldots ,N}}{\text{arg}\;\text{max}}K\left(w\right),\;K\left(w\right)≔\sum_{i=1}^{N}v_{i}w_{i}+\int_{y\in \Omega }\;\underset{i=1,\ldots ,N}{\text{min}}\left[c\left(y,x_{i}\right)-w_{i}\right]\;dy$$

The objective function K(w) is concave as the sum and minimum of functions that are linear with respect to *w*. Its gradient simply reads

$$\nabla K\left(w\right)={\left(v_{i}-|L_{i}|\right)}_{i=1,\ldots ,N}$$

where $L_{i}$ is defined by Eq. 1 for an arbitrary set of potentials. This implies that the optimality conditions $|L_{i}|=v_{i}$ are satisfied at the maximum of K, which is well-defined if the sum of the target volumes *v_i_* does not exceed that of the domain Ω and if the cost functions $c_{i}:y\mapsto c\left(y,x_{i}\right)$ satisfy mild technical assumptions: notably, distinct indices i≠j should correspond to distinct cost functions $c_{i}\neq c_{j}$.

To solve this maximization problem, as in (*52*), we rely on a classical quasi-Newton method, namely, the L-BFGS-B algorithm. At each time step, the stopping criterion for this optimization algorithm is when the average volume deviation (between the computed volumes and the theoretical volumes) falls below 1%. Except for rare isolated events, this is always the case in all the experiments presented.

In a discretized domain $\Omega =\left\{y_{1},\ldots ,y_{M}\right\}$ with *M* large, the most expensive part of the computation of the objective function is the minimum operation over the *i*-indices of the *N* × *M* matrix ${\left[c\left(y_{j},x_{i}\right)-w_{i}\right]}_{i,j}$ followed by a sum over the *j*-indices approximating the integral. To handle arbitrary cost functions and fine discretizations with $M∼10^{6}$ voxels, we rely on the semi-symbolic lazy tensor framework introduced by the KeOps and GeomLoss libraries (*63*). This lets our plain Python code perform scalable geometric computations on the GPU, with automatic differentiation and without memory overflows.

The assignment of each voxel to one of the **x***_i_* is then given by the following map, which in optimal transport corresponds to a discretized variant of the Monge transport map (*57*)

$$T:{\left\{y_{j}\right\}}_{j\in \left\{1,\ldots ,M\right\}}\to {\left\{x_{i}\right\}}_{i\in \left\{1,\ldots ,N\right\}},\;y_{j}\mapsto x_{j^{*}}$$

with

$$j^{*}≔\underset{i\in \left\{1,\ldots ,N\right\}}{\text{arg}\;\text{max}}\left\{c\left(y_{j},x_{i}\right)-w_{i}\right\}$$

Semi-discrete optimal transport on a discretized grid and the voxel assignment procedure is illustrated is dimension 1 in fig. S1.

### Software and hardware

All the simulations presented in this article run on an Nvidia RTX A6000 GPU card. We use NumPy, PyTorch, and KeOps for our simulations while relying on Matplotlib, PyVista, and Paraview for visualizations.

### Cost normalization

We assume that the domain denoted by Ω is connected and bounded, with total volume normalized to 1 and typical length *L* = 1. The cell positions **x***_i_* and the other quantities are assumed to be properly adimensionalized with respect to this length scale.

Unless otherwise specified, the cost function *c* is implicitly normalized so that, given a shape $S_{0}\subset \Omega$ corresponding to a level set of *c*, with centroid at **x**_0_, and a normalizing factor denoted by λ in the parameter table,

$$\int_{S_{0}}c\left(x,x_{0}\right)dx=\lambda |S_{0}|$$

In other words, the total cost of the shape $S_{0}$ is a fraction of its volume. When *c* is the power cost $c\left(x,y\right)={\lambda_{\alpha }}^{-1}{|x-y|}^{\alpha }$, we consider a fixed radius *R*_0_ and thus take in dimension *d*,

$$\lambda_{\alpha }=\frac{\lambda^{-1}d}{\alpha +d}R_{0}^{\alpha }$$

In this case, we always take *R*_0_ as the average radius of the particles. For the anisotropic costs (ellipsoidal and spherocylinder costs), the cost of a particle with aspect ratio *r* is normalized to be equal to a fraction λ of the volume of the same shape with aspect ratio *r* associated to a given small axis denoted by *b* in the parameter table.

With this normalization choice, the incompressibility force defined (Eq. 3) scales like $|\nabla_{x_{i}}T_{c}\left(\hat{\mu }\right)|\propto R_{0}$. We thus implicitly normalize the gradient descent step in Eq. 3 as $\tau \equiv \tau /R_{0}$ so that the gradient descent motion is independent of the size *R*_0_ of the particles. In other words, if one reduces or increases the volume of the particles, one simulates the same system but in a respectively larger or smaller domain.

### Boundary conditions

In all experiments, there is either no boundary conditions or periodic boundary conditions. Without boundary conditions, we note that the only important quantity to consider is the cost function c(x,y)=c(y−x), which is always a function of the connecting vector **y** − **x** between two points $x,y\in \Omega ={\left[0,1\right]}^{d}$. In the periodic case, we implicitly replace this vector by its “periodized” version obtained by taking the shortest connecting vector between two representative points of **x** and **y** in the full space. More precisely, by denoting $z=\left(z^{1},\ldots ,z^{d}\right)$ the components of a vector **z**, its periodized version can be obtained by applying the following transformation on each component *z^k^*

$$z^{k}\mapsto z^{k}+\chi_{z^{k}<-1/2}-\chi_{z^{k}>1/2}$$

where $\chi_{z^{k}<-1/2}=1$ if $z^{k}<-1/2$ and 0 otherwise. To alleviate notations, we only write the cost function and other quantities assuming no boundary conditions, but we implicitly use the periodized version of the vectors to handle periodic boundary conditions. Besides, positions are simply computed modulo 1 when needed.

### Anisotropic shapes

The PCA of an anisotropic shape $L_{i}$ is computed in a discretized setting as the empirical covariance matrix

$${\hat{\Sigma }}_{i}=\frac{1}{N_{i}-1}\sum_{y\in L_{i}}\left(y-x_{i}\right){\left(y-x_{i}\right)}^{T},\;N_{i}≔\#\left\{y\in L_{i}\right\}$$

The covariance matrix defining the orientation of a particle in Figs. 3C and 2B is simply defined as

$$\Sigma_{i}=\frac{1}{\text{det}{\left({\hat{\Sigma }}_{i}\right)}^{1/d}}{\hat{\Sigma }}_{i}$$

The direction of motion **n***_i_* is defined as the normalized eigenvector associated to the largest eigenvalue of the matrix ${\hat{\Sigma }}_{i}$ (with the sign correctly chosen to avoid flipping the direction of motion).

### Surface meshing and curvature computation

The computation of interfacial forces (Eq. 13) requires to integrate quantities along the boundary of each Laguerre cell. Since our optimal transport algorithm outputs volumetric data, we use the fast isocontouring algorithm SurfaceNets (*77*), which is integrated in VTK (*78*) to convert at each time step the Laguerre cells into mesh data. We then rely on the routines already implemented in VTK and PyVista (*79*) to compute the normal and curvature of each mesh element. For better numerical stability we filter the mesh elements for which the normal direction is not well defined.

## Supplementary Materials

### The PDF file includes:

Supplementary Text Figs. S1 to S9 Tables S1 and S2 Legends for movies S1 to S23 References
