# Supplementary material for: Multicellular simulations with shape and volume constraints using optimal transport
Source: Sci Adv. 2026 Jul 10;12(28):eadv2781. doi: 10.1126/sciadv.adv2781 (PMC13353413; doi:10.1126/sciadv.adv2781)
Supplement: Supplementary file 1 — Supplementary Text Figs. S1 to S9 Tables S1 and S2 Legends for movies S1 to S23 References [file sciadv.adv2781_sm.pdf]

Science Advances
AAAS

Supplementary Materials for

Multicellular simulations with shape and volume constraints using optimal transport

Antoine Diez and Jean Feydy

Corresponding author: Antoine Diez, antoine.diez@riken.jp

Sci. Adv. **12**, eadv2781 (2026)
DOI: 10.1126/sciadv.adv2781

The PDF file includes:

Supplementary Text
Figs. S1 to S9
Tables S1 and S2
Legends for movies S1 to S23
References

Other Supplementary Material for this manuscript includes the following:

Movies S1 to S23

Supplementary text

Supplementary experiments

The transition from a fluid to a solid phase as shown in the main text plays an important role in the study of congestion effects in crowd modeling. Here, the active Brownian particle model can be adapted to an evacuation situation where all particles (modeling pedestrians) move towards a single exit point. When the pedestrians are assumed to have the ability to deform ($\alpha = 0.5$), the crowd behaves as a fluid and safely evacuates the room. When pedestrians are modeled by hard-spheres ($\alpha = 8$), congestion effects appears, typically materialized by the formation of an arch around the exit point (*2*), which is stable even in the presence of noise (Fig. S2 and SM Videos 15,16).

In the main text, we consider a homogeneous population of active deformable particles with different deformability properties. It is also possible to mix soft and hard particles by assigning different costs to different populations. As an example, we consider a 2D falling soft-sphere experiment. In a crowded situation, there is a competition between the incompressibility force $-\tau\nabla_{\mathbf{x}_i}\mathcal{T}_c$ and the gravity-like force $\mathbf{F}_i^{\mathrm{point}} = -F_0\mathbf{e}_z$. When the population is homogeneous, we recover the results shown in the main text in particular a hard-sphere packing configuration. In this particular situation, when α is small, the particles manage to squeeze their way by adopting elongated columnar shapes (Fig. S3).

To study the effect of heterogeneity we now consider the same situation but with two equal populations associated to two values $\alpha = 1$ and $\alpha = 2$. In addition, we also consider two different values for the gradient-step parameter τ, which plays the role as an inertia parameter: although the particles with $\alpha = 1$ are always softer, they appear as heavier when τ is small and lighter when τ is big. It leads to a sorting phenomena where lighter particles are pushed on top (Fig. S4 and SM Videos 20,21,22). It is always easier for softer particles to squeeze their way down, which leads to non-convex or elongated shapes.

Biological cells are often regarded as similar to soap bubbles. Laguerre tessellation have actually already been used to model bubbles and foams in computer graphics (*55*) but with a simpler model compared to the model presented in the main text. Compared to biological cells, bubbles are simpler because they must satisfy the Plateau conditions which impose a constant curvature and a 120° angle between two bubbles of equal size. In our model, they can be enforced by setting all surface

tension parameters to 1. Simulating bubble interactions is however challenging as they can have very heterogeneous volumes and their number is not conserved in time due to frequent fusion, explosion, creation events. In particular, traditional DCM would need to deal with many rearrangements and topological transitions. Our model does not has such flaws. As an illustrative example we simulate a very simple system of bubbles. We assume that bubbles are generated randomly within a fluid which occupies the bottom half of a square domain. Due to gravity and buoyancy forces, the bubbles stay at the interface between the fluid and the air. We add a small force which pushes the bubbles towards the boundary of the domain as in (*55*). We assume that bubbles can explode randomly with a higher probability when they are larger. Finally, two bubbles can randomly fuse when they are in contact, which creates a single bubble with a volume which is the sum of the two fused bubbles. The simulation is shown in SM Video 23 and Fig. S5.

Table S2 gathers the modeling and numerical parameters for the supplementary experiments.

Optimal transport point of view

We gather here some important definitions and results that may be useful for a better understanding of the main text. Optimal transport theory is by now well-established and we refer to (*58*) and the references therein for a deeper introduction to the subject from a mathematical perspective and to (*56, 57*) for a focus on numerical methods and applications in data sciences and computational geometry.

Historically, Monge considered the problem of transporting sand piles located at some positions $\mathbf{y}_1, \dots, \mathbf{y}_M \in \Omega \subset \mathbb{R}^d$ and with respective masses $\beta_1, \dots, \beta_M$, to some target positions $\mathbf{x}_1, \dots, \mathbf{x}_N \in \Omega$ that should respectively receive a volume $\alpha_1, \dots, \alpha_N$ of sand. The total volume $\sum_j \beta_j = \sum_i \alpha_i = 1$ is classically normalized to 1. The transport of a sand grain between the locations $\mathbf{y}$ and $\mathbf{x}$ comes with a cost $c(\mathbf{y}, \mathbf{x})$; the optimal transport problem is to find an optimal way of moving all of the sand at a minimal cost. When $N = M$ and $\alpha_i = \beta_j = 1/N$, this problem can be seen as a *matching* problem, which consists in finding the one-to-one map (or equivalently a permutation of the set $\{1, \dots, N\}$)

$$T : \{\mathbf{y}_1, \dots, \mathbf{y}_N\} \to \{\mathbf{x}_1, \dots, \mathbf{x}_N\},$$

that minimizes the total transport cost

$$\mathcal{T}_c := \frac{1}{N}\sum_{i=1}^{N} c(\mathbf{y}_i, T(\mathbf{y}_i)).$$

In the general case with $N \neq M$ and arbitrary volumes, such a *Monge map* may not exist since we may have to split the sand piles and send their different parts to different locations. The optimal transport problem then only makes sense for the more general notion of *transport plan* introduced by Kantorovich. In this setting, a transport plan is a $N \times M$ matrix $\pi = (\pi_{ij})$ whose coefficients $\pi_{ij} \geq 0$ indicate how much of the sand pile j is sent to the location i. Consequently, the transport plan π should satisfy the constraints

$$\forall j,\ \sum_{i=1}^{N} \pi_{ij} = \beta_j, \quad \forall i,\ \sum_{j=1}^{M} \pi_{ij} = \alpha_i.$$

The optimal transport problem then consists in finding the matrix π that satisfies this constraints and minimizes the total transport cost

$$\mathcal{T}_c := \sum_{i=1}^{N}\sum_{j=1}^{M} \pi_{ij} c(\mathbf{y}_j, \mathbf{x}_i).$$

Going further, this problem can be reformulated using measure theory. The sand piles and target locations are respectively modeled by the discrete probability measures

$$\nu = \sum_{j=1}^{M} \beta_j \delta_{\mathbf{y}_j}, \quad \mu = \sum_{i=1}^{N} \alpha_i \delta_{\mathbf{x}_i}. \tag{S1}$$

The Monge problem corresponds to the minimization problem

$$\inf_{T:\Omega\to\Omega} \left\{ \int_\Omega c(\mathbf{y}, T(\mathbf{y}))\nu(\mathrm{d}\mathbf{y}) \,\middle|\, T\#\nu = \mu \right\}, \tag{S2}$$

where we recall that the *push-forward* measure $T\#\nu$ is defined as the measure on Ω such that

$$T\#\nu(\mathscr{B}) = \nu(T^{-1}(\mathscr{B})),$$

for all open sets $\mathscr{B}$.

The Kantorovich problem corresponds to the minimization problem

$$\inf_{\Pi\in\mathcal{P}(\Omega\times\Omega)} \left\{ \int_\Omega\int_\Omega c(\mathbf{y}, \mathbf{x})\Pi(\mathrm{d}\mathbf{y}, \mathrm{d}\mathbf{x}) \,\middle|\, \Pi_1 = \nu,\ \Pi_2 = \mu \right\}, \tag{S3}$$

where $\mathcal{P}(\Omega \times \Omega)$ denotes the set of probability measures on $\Omega \times \Omega$ and Π_1 and Π_2 are the first and second marginals of $\Pi \in \mathcal{P}(\Omega \times \Omega)$.

Although it can be checked that with the choice (S1), the Monge and Kantorovich problems reduce to the optimization problems introduced above, the formulations (S2)-(S3) are much more general and are not restricted to the case of discrete measures μ, ν. In particular, μ and/or ν can be continuous measures (defined by their probability density function). In this case Brenier, Gangbo, McCann, Caffarelli and others have proved the following fundamental theorem.

Theorem 1 *If the source measure* ν *is a continuous measure and under some assumptions on the cost function* c*, then the Monge problem* (S2) *has a unique solution* T*.*

In our approach, we consider the semi-discrete case where $\mu = \sum_i \alpha_i \delta_{\mathbf{x}_i}$ is a discrete measure and $\nu = \text{Leb}$ is a continuous measure, here the Lebesgue measure. In this case, a direct application of the previous theorem shows that the optimal transport problem is equivalent to the computation of a partition of the space $\Omega = \cup_i \mathcal{S}_i$ into the disjoint sets:

$$\mathcal{S}_i := T^{-1}(\{\mathbf{x}_i\}).$$

Moreover, a simple computation shows that $\nu(\mathcal{S}_i) = \alpha_i$. The fundamental theorem of semi-discrete optimal transport theorem, which is the theoretical basis of our work, can be found in (*59, 76*). It proves that, for a large class of cost function, our problem always has a unique solution.

Theorem 2 *Let* $\hat{\mu} = \sum_{i=1}^{N} \nu_i \delta_{\mathbf{x}_i}$ *be a discrete probability measure on* Ω*. Let* c *be a cost function such that for all* i*,* $c_i : \mathbf{x} \in \Omega \mapsto c(\mathbf{x}, \mathbf{x}_i) \in [0, +\infty)$ *belongs to* $C^{1,1}(\Omega)$ *and* $\mathbf{y} \mapsto \nabla_{\mathbf{x}} c(\mathbf{x}, \mathbf{y})$ *is injective for all* $\mathbf{x} \in \Omega$*. Then the Monge problem* (S2) *has a unique solution* T *given by*

$$T : \Omega \to \{\mathbf{x}_i\}_i, \;\; \mathbf{x} \in \mathcal{L}_i \mapsto \mathbf{x}_i$$

where the partioning sets $(\mathcal{L}_i)_i$ *are the Laguerre cells defined by* (1)*. The intersection of two Laguerre cells has zero Lebesgue measure and the weights* $w_i \in \mathbb{R}$*, called Kantorovich potentials, are uniquely defined such that for all* $i \in \{1, \ldots, N\}$*,* $|\mathcal{L}_i| = \nu_i$*.*

A graphical illustration of the discrete, continuous and semi-discrete problems is shown in Fig. S6.

Coarse-grained analysis

There is a fundamental duality between agent-based and continuum Partial Differential Equations (PDE) models in mathematical biology. Each description has its own strengths and weaknesses. Typically, agent-based models provide the finest level of details and modeling freedom and are naturally adapted to in silico computations with potentially stochastic components. However, they provide little room for theoretical mathematical analysis due to their complexity and usually high-dimensional nature. On the contrary, continuum PDE models provide a synoptic point of view at a statistical scale, benefit from all the analytical mathematical machinery, are often closer to physical laws and can eventually lead to much stronger "proved" conclusions. Bridging the gap between the two descriptions in a rigorous mathematical manner is a fundamental issue in mathematical physics and mathematical biology, but its feasibility strongly depends on the modeling framework (*74*). Point-particle systems are classically the easier to coarse-grain, as they can often be written in a statistical physics framework for which many theoretical results are now available (*14*). Particles with a shape, regardless of the description (vertex models, cellular automata, phase-fields etc), seem much more difficult to handle, since no universal or natural continuum limit and scaling can be easily deduced. The framework introduced in the present article is somehow hybrid, it can be seen as an intrinsically agent-based model (4) but allows a control on the shape and volume exclusion parameters. Formal computations (at this stage) and earlier works in computational optimal transport suggest that this underlying point-particle description can naturally lead to coarse-grained continuum PDE models.

Let us consider a simplified version of the equations of motion (4), with no surface interaction and independent Brownian noises:

$$\mathrm{d}\mathbf{x}_i = b(\mathbf{x}_i, \hat{\mu})\mathrm{d}t - \tau\nabla_{\mathbf{x}_i}\mathcal{T}_c\mathrm{d}t + \sqrt{2\sigma}\mathrm{d}\mathbf{B}^i_t. \tag{S4}$$

The force term $\mathbf{F}_i^{\mathrm{point}} = b(\mathbf{x}_i, \hat{\mu})$ is assumed to depend on the empirical measure

$$\hat{\mu} = \frac{1}{N}\sum_{j=1}^{N}\delta_{\mathbf{x}_j},$$

which encompasses for instance, all binary interaction models. Without the incompressibility force, this system is known as a McKean-Vlasov system and has been studied in great details since

the seminal works of McKean and Kac, see the review (*14*). The main idea, which originates from statistical physics, is to consider the limit of the sequence of random empirical measures $\hat{\mu} \equiv \hat{\mu}_N := \frac{1}{N}\sum_{i=1}^{N} \delta_{\mathbf{x}_i}$ when $N \to +\infty$. When $\tau = 0$ and under some assumptions on b, it can be shown that this sequence has a deterministic limit $\hat{\mu}_N \to f$ which is the solution of the (nonlinear) Fokker-Planck equation

$$\partial_t f = -\nabla_{\mathbf{x}} \cdot (b(\mathbf{x}, f) f) + \Delta_{\mathbf{x}} f.$$

The solution f is the probability distribution of the state of a single typical particle and thus models the system at a statistical scale. Many important mathematical results, which are relevant in biology, have been obtained with this method, see for instance (*14*) for a review of applications, in particular in collective dynamics.

Coming back to the Eq. (S4), a first difficulty comes from the fact that the incompressibility force $\nabla_{\mathbf{x}_i}\mathcal{T}_c(\hat{\mu})$ does not appear as a simple function of $\hat{\mu}$ due to the gradient in $\mathbf{x}_i$. To overcome this issue, we recall the notion of first variation of a functional $F : \mathcal{P}(\Omega) \to \mathbb{R}$ on the space of probability measures $\mathcal{P}(\Omega)$: this is the function $\frac{\delta F}{\delta \mu}(\mu) : \Omega \to \mathbb{R}$ defined such that

$$\frac{\mathrm{d}}{\mathrm{d}h} F(\mu + h\chi)\Big|_{h=0} = \int_\Omega \frac{\delta F}{\delta \mu}(\mu)\mathrm{d}\chi,$$

for every measure χ such that $\mu + h\chi \in \mathcal{P}(\Omega)$ for small enough h. With this notion, it is possible to apply the chain rule to compute the gradient

$$\nabla_{\mathbf{x}_i}\mathcal{T}_c(\hat{\mu}) = N^{-1}\nabla\frac{\delta \mathcal{T}_c}{\delta \mu}(\hat{\mu})(\mathbf{x}_i),$$

where the gradient on the right-hand side is the usual gradient of a real-valued function on $\mathbb{R}^d$. Thus taking the scale $\tau \equiv \tau_N = N\tau_0$, Eq. (S4) can be rewritten,

$$\mathrm{d}\mathbf{x}_i = b(\mathbf{x}_i, \hat{\mu})\mathrm{d}t - \tau_0\nabla\frac{\delta \mathcal{T}_c}{\delta \mu}(\hat{\mu})(\mathbf{x}_i)\mathrm{d}t + \sqrt{2\sigma_i}\mathrm{d}\mathbf{B}_t^i. \tag{S5}$$

This latter equation enters the classical framework and leads to the formal mean-field limit

$$\partial_t f = -\nabla \cdot (b(\mathbf{x}, f) f) + \tau_0 \nabla \cdot \left(\nabla\frac{\delta \mathcal{T}_c}{\delta \mu}(f) f\right) + \Delta f. \tag{S6}$$

At this stage, it remains to identify the first variation of the transport cost $\mathcal{T}_c$. We first note that if f satisfies Eq. (S6), it should have a density with respect to the Lebesgue measure. We also assume that the cost is of the form $c(\mathbf{x}, \mathbf{y}) = \ell(\mathbf{x} - \mathbf{y})$ for a strictly convex function $\ell : \mathbb{R}^d \to [0, +\infty)$. Since

the work of Brenier, McCann, Gbangbo, Cafferelli and others, it is well-known that the Monge problem $T\#f = \mathrm{Leb}$ has a unique solution which is of the form

$$T(\mathbf{x}) = \mathbf{x} - (\nabla\ell)^{-1}(\nabla\Phi(\mathbf{x})),$$

where Φ is a Kantorovich potential which satisfies the Monge-Ampère equation (*67*)

$$|\det(\nabla T)| = f.$$

Then it can be shown (*58*) that

$$\frac{\delta\mathcal{T}_c}{\delta\mu}(f) = \Phi.$$

Consequently, and after a few more computations, Eq. (S6) can be rewritten as a coupled system of Fokker-Planck-Monge-Ampère equations

$$\partial_t f = -\nabla\cdot(b(\mathbf{x}, f)f) + \tau_0\nabla\cdot(\nabla\Phi f) + \Delta f. \tag{S7a}$$

$$\det\left(\mathrm{Id} - \nabla^2\ell^*(\nabla\Phi)\nabla^2\Phi\right) = f, \tag{S7b}$$

where ℓ^* denotes the Legendre transform of ℓ defined by

$$\ell^*(\mathbf{y}) = \sup_{\mathbf{x}\in\mathbb{R}^d}\{\mathbf{x}\cdot\mathbf{y} - \ell(\mathbf{x})\}.$$

For the L^2 cost $c(\mathbf{x}, \mathbf{y}) = \frac{1}{2}|\mathbf{x} - \mathbf{y}|^2$, it holds that $\ell^* = \ell$ and thus $\nabla^2\ell^*$ is the identity and Eq. (S7b) reduces to

$$\det(\mathrm{Id} - \nabla^2\Phi) = f.$$

With the notable exception of (*68*) which discusses connection with the Euler equation, the general equation (S7) is not common in the literature and, to the best of our knowledge, it is new in a mathematical biology framework. Its mathematical analysis and numerical treatment remain largely open, as well as the study of different scaling limits and the influence of the cost function. This may represent a novel rigorous approach to derive continuum PDE models from systems of particles with volume interactions. Connections with continuum model of crowd motion (*49, 75*) are also natural although the authors of these works follow a different path than mean-field theory.

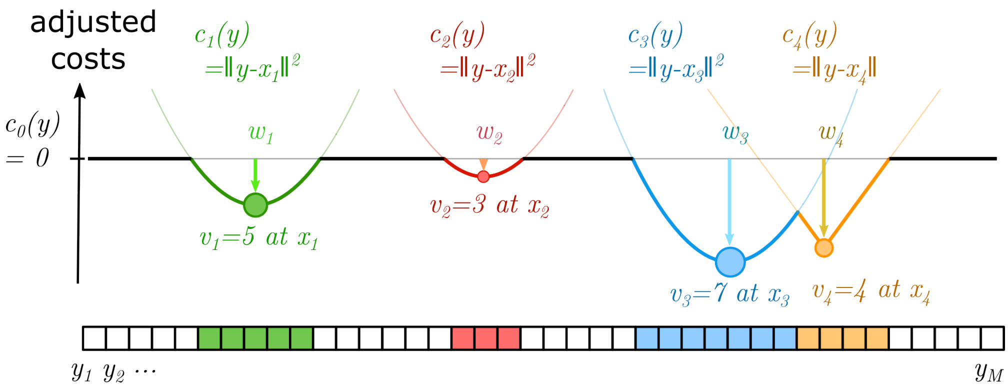

Figure S1: **Semi-discrete optimal transport on a grid in dimension 1 with four particles.** Each voxel is assigned to the minimal adjusted cost. The potentials w_i are adjusted to satisfy the volume constraints.

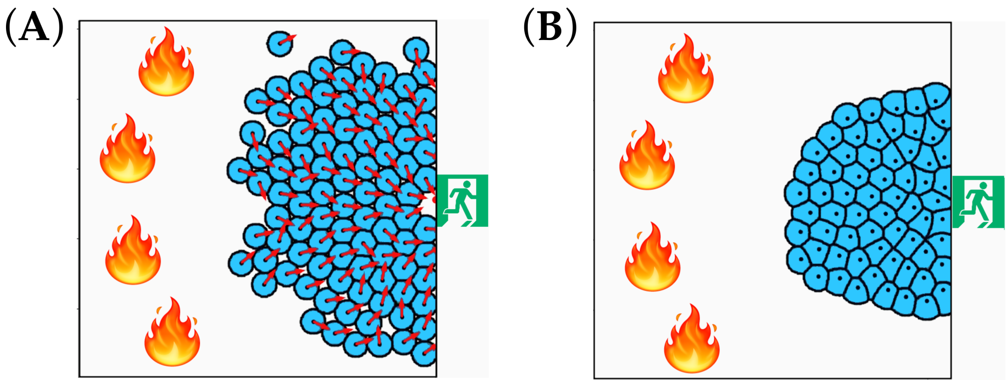

Figure S2: **Crowd simulations. (A)** $\alpha = 8$. Formation of a stable arch around the exit point when deformations are not allowed. **(B)** $\alpha = 0.5$. The crowd is able to leave the room when deformations are allowed. See also SM Videos 15,16.

Supplementary figures

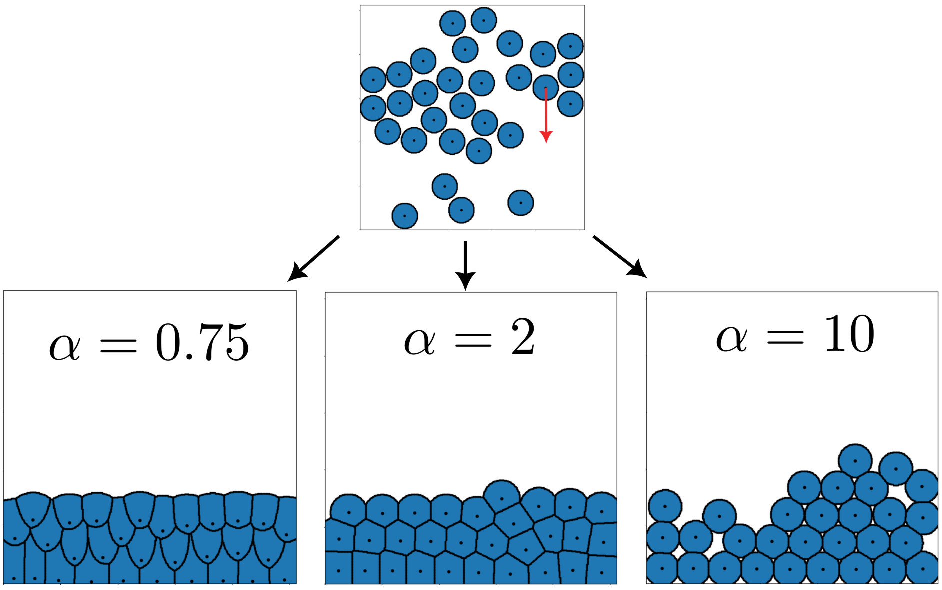

Figure S3: **Falling soft spheres.** Initial configuration (top) and equilibrium configuration (bottom) of a system of falling soft spheres for three values of the deformation parameter α. See also SM Videos 17,18,19.

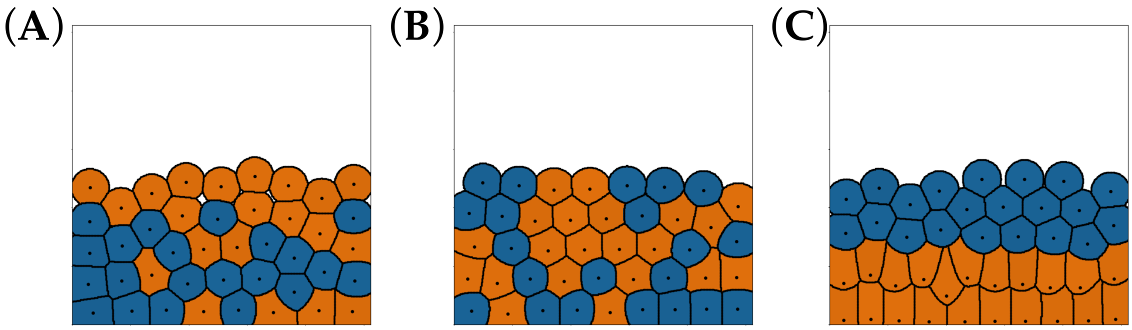

Figure S4: **Final configuration of a mixed system of 30 falling spheres with different weights and softness.** The 15 blue spheres are harder ($\alpha = 2$) and the orange spheres are softer ($\alpha = 1$). The gradient descent step of the blue spheres is fixed to $\tau_b = 3$ and both the orange and blue spheres are subject to the same downward force with magnitude $F_0 = 0.4$. The gradient descent step of the orange particles τ_o is analogous to the inverse of a mass. (a) Light orange spheres ($\tau_o = 8$) (b) Same weight ($\tau_o = 2$) (c) Heavier orange spheres ($\tau_o = 1$). See also SM Videos 20,21,22.

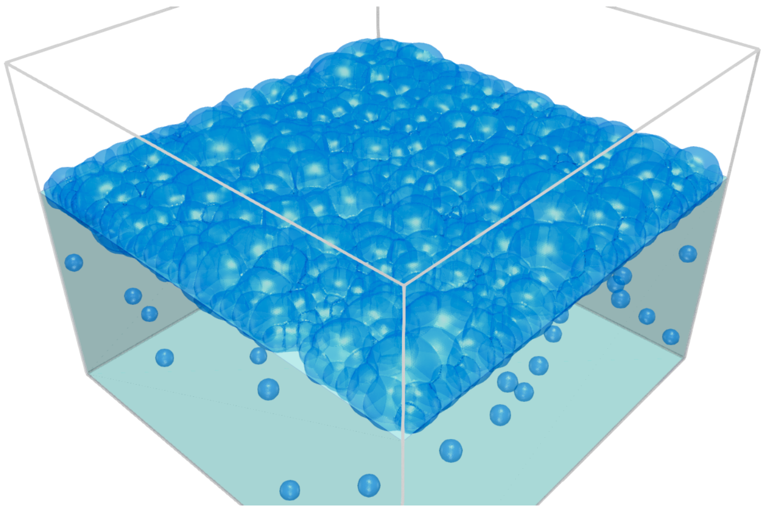

Figure S5: **Bubble simulations.** See also SM Video 23.

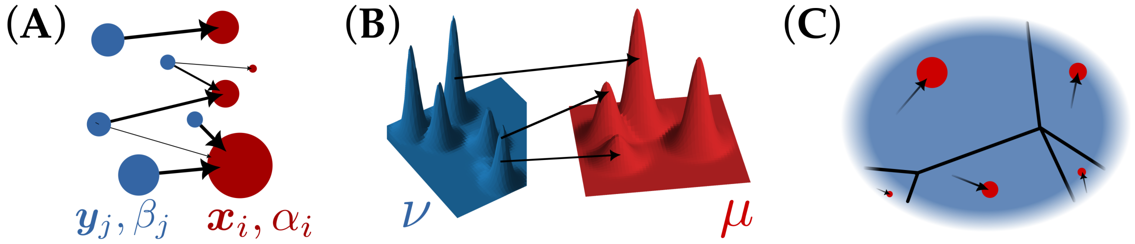

Figure S6: **Discrete, continuous and semi-discrete optimal transport. (A)** A discrete optimal transport problem is a matching point problem but which may require mass splitting in which case there is no Monge map. **(B)** If the source measure has a density, then there is a Monge map. **(C)** In the semi-discrete case, the target measure is discrete and the Monge map defines a partition of the space.

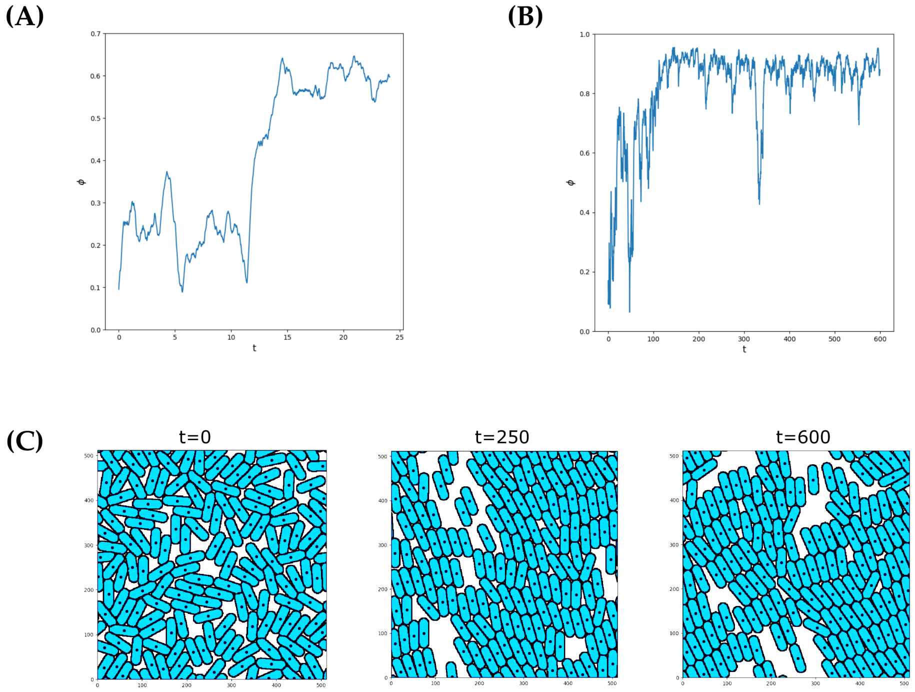

Figure S7: **Nematic order parameter over time in the simulation of rod-shape particles. (A)** Related to Fig. 2B **(B)** Nematic order parameter over an extended simulation time (T = 600) for a smaller but denser system (N = 150, V = 0.8, α = 8). The other parameters are the same as Fig. 2B. **(C)** Snapshots of the simulation (B) at three time points showing the persistence of orientational order.

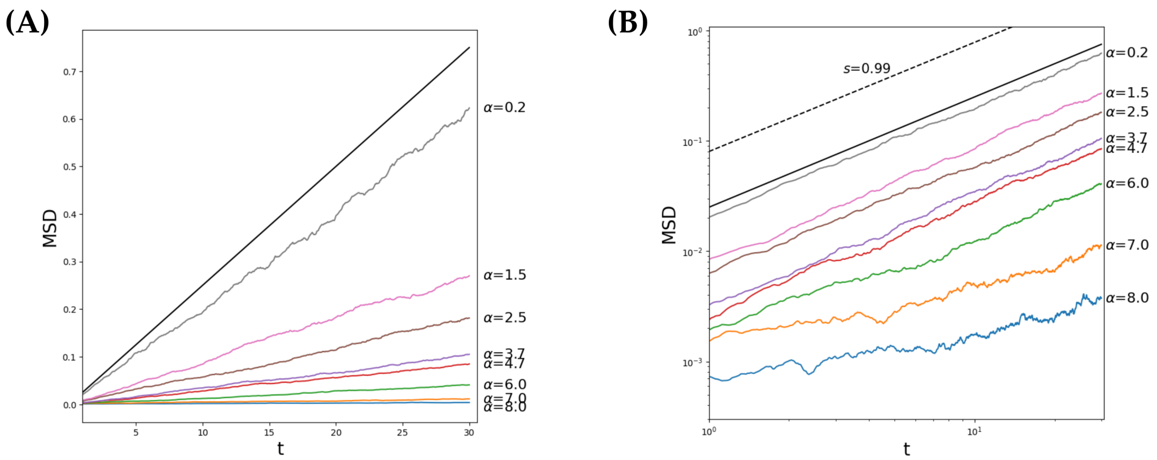

Figure S8: **MSD for Active Brownian Particles with various deformability parameters. (A)** Linear scale until time $t = 30$ for eight deformability parameters α and $c_0 = 0.5$. The solid black line has the theoretical slope for non-interacting ABM. **(B)** Log-log scale plot for $t \in (1, 30)$. The average anomalous diffusion coefficient computed for the fluid-like systems in the range $\alpha \leq 6$ is $s = 0.99$ (dashed line).

Supplementary tables

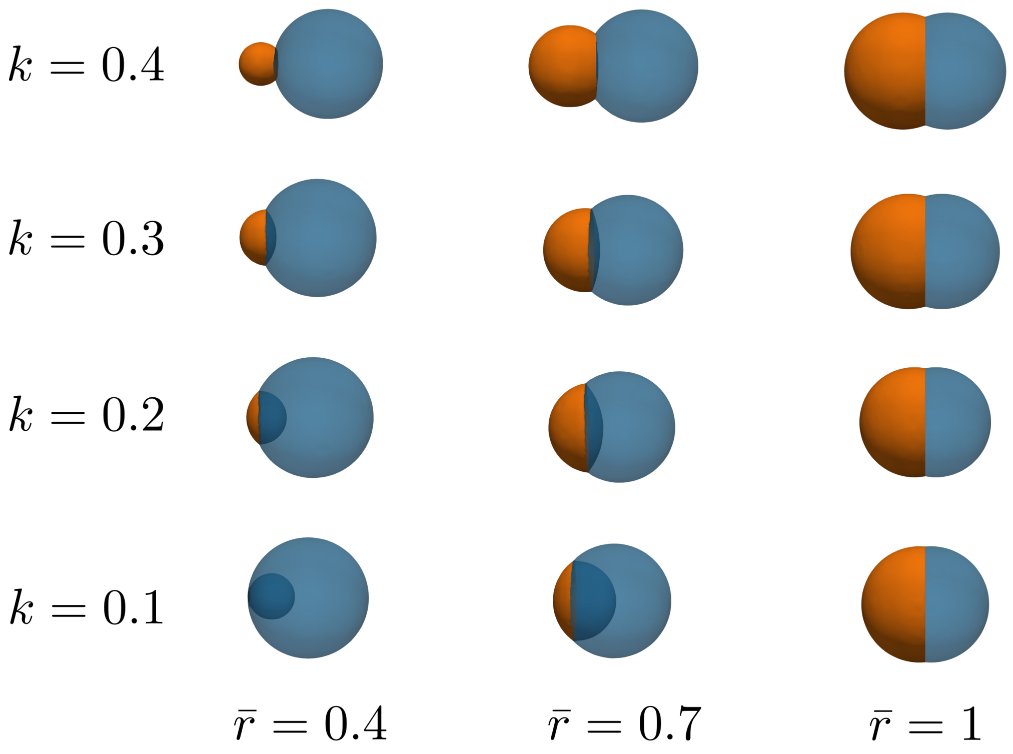

Figure S9: **Equilibrium configurations for two 3D cells with different volumes.** Simulation of two 3D Laguerre cells with different volume v_o and v_b (resp. orange and blue) until an equilibrium configuration of the model (13) is reached. The blue cell is displayed with lower opacity. We define the ratios $k = \frac{\eta_{ob}}{2\gamma_o}$ and $\bar{r} = (v_o/v_b)^{1/3}$. The fixed parameters are $\gamma_o = \gamma_b = 7$, $\gamma_{ob} = 1$ and we set $\gamma_{oo} = \gamma_{bb} = \eta_{oo} = \eta_{bb} = 0$ since there is only one orange/blue interface.

Table S1: **Modeling and numerical parameters.** This table gathers all the detailed modeling and numerical parameters used in the experiments. When the parameters differ between blue and orange particles, they are identified respectively by the subscripts b and o. The parameters λ and b refer to the cost normalization procedure with $\lambda = 1$ if not specified. When a single value of V is given, it corresponds to the total volume of the N particles which are all assumed to have the same volume V/N. The space domain Ω is discretized with M voxels in dimension d. The value Δt corresponds to the time discretization step in the Euler method. The vector $\mathbf{e}_z$ denotes the unit vector pointing upward. The operator $\mathsf{P}_{\mathbf{n}^\perp}$ denotes the projection on the orthogonal of a unit vector $\mathbf{n}$ and $\mathbf{B}_t^i$ is a Brownian motion. The point $\mathbf{x}_{\text{exit}} = (1, 0.5)^{\mathrm{T}}$ denotes the exit point in Fig. S2. The notation $x = (x_1, x_2, x_3)$ indicates the values for the respective figures indexed by (a, b, c) or from left to right. The notation $x \in (x_1, x_2)$ indicates that the value of x is sampled uniformly (randomly if not indicated) within the interval (x_1, x_2) for each particle.

Experiment	cost	$\mathbf{F}^{\text{point}}$	$\mathbf{F}^{\text{surf}}$	τ	other	N	V	Δt	$M^{1/d}$
Fig. 3A	L^2<br>$\lambda = 0.25$	$-0.5\mathbf{e}_z$	0	3	NR	50 to 2,000	0.5	0.005	512
Fig. 3B	L^2<br>$\lambda = 0.1$	0	0	10	growth rate: $3\gamma v_0$<br>$\gamma \in (0.5, 2)$ random<br>division rate: 3	1 to 50,000	target volume:<br>$v_0 = \frac{4}{3}\pi 0.15^3$	0.002	400
Fig. 3C	(5)<br>$\lambda \in (3, 4)$,<br>$b = 1$	$0.3\mathbf{n}_i$	0	42	aspect ratio: 1 to 4	10 to 10,000	$V = 0.8$<br>random with ratio 1/5	0.005	64 to 512
Fig. 2B	(6)-(7)<br>$\alpha = 3.5$	$0.3\mathbf{n}_i$	0	0.14	aspect ratio: 3	300	0.65	0.002	512
Fig. 4	(8) $\alpha \in (0.2, 8)$<br>$\lambda = 0.1$	$c_0\mathbf{n}_i$<br>$\dot{n}_i = \sqrt{2\sigma}\mathsf{P}_{\mathbf{n}_i^\perp} \circ \dot{\mathbf{B}}_t^i$	0	3	$\sigma = 20$<br>$c_0 \in (0.02, 2)$	250	0.94	0.002	512
Fig. 2C	(11)	0	0	1	$\beta = 0.02$, (12)<br>u Gaussian with variance 0.2^2	10	0.1	0.01	512
Fig. 5A	(13)	0	(13)	0	$\overline{\eta} = 3$, $k_2 = k_{12} = 0.4$, $\gamma_2 = 10$<br>$\overline{\gamma} = 1/3$, $\overline{k} = (0.06, 0.21, 0.67, 1, 2)$<br>$\overline{\gamma} = 1$, $\overline{k} = (0.17, 0.67)$	$N_o = 60$<br>$N_b = 60$	0.27	0.001	256
Fig. 5B	(13)	0	(13)	0	$\overline{\eta} = 0.3$, $k_2 = k_{12} = 0.4$, $\gamma_2 = 10$<br>$\overline{k} = 0.5$, $\overline{\gamma} = (1, 1.3, 3.1, 13.3)$<br>$\overline{k} = 1$, $\overline{\gamma} = (1, 1.5, 6.7)$<br>$\overline{k} = 2$, $\overline{\gamma} = (1.25, 3.3)$	$N_o = 60$<br>$N_b = 60$	0.27	0.001	256
Fig. 5C	(13)	0	(13)	0	$\overline{\eta} = 3$, $\overline{\gamma} = 2$, $\overline{k\gamma\eta} = 0.8$	$N_o = 64$<br>$N_b = 64$	0.29	0.0003	256

Table S2: Modeling and numerical parameters for the Supplementary experiments

Experiment	cost	$\mathbf{F}^{\text{point}}$	$\mathbf{F}^{\text{surf}}$	τ	other	N	V	Δt	$M^{1/d}$
Fig. S2A,B	(8), $\alpha = (0.5, 8)$ $\lambda = 0.1$	$0.4\mathbf{n}_i$ $\mathbf{n}_i = (\mathbf{x}_i - \mathbf{x}_{\text{exit}})/\lvert\mathbf{x}_i - \mathbf{x}_{\text{exit}}\rvert$	0	3	$\sigma = 0.2$	111	0.42	0.001	512
Fig. S3	(8) $\alpha = (0.75, 2, 10)$ $\lambda = 0.125$	$-0.4\mathbf{e}_z$	0	1.5	NR	30	0.3	0.001	512
Fig. S4A,B,C	(8), $\alpha_b = 2$, $\alpha_o = 1$ $\lambda = 0.125$	$-0.4\mathbf{e}_z$	0	$\tau_b = 3$ $\tau_o = (1, 3, 8)$	NR	$N_b = N_o = 21$	0.5	0.001	512

List of Supplementary Videos

The following supplementary videos can be found on Figshare

`https://doi.org/10.6084/m9.figshare.25240669`

or on the documentation website alongside with the related Python scripts (and additional simulations not shown in the present article). In all the videos, the volume of the domain is normalized to 1 but the ticks on the axes are labeled between 1 and M (the number of discrete voxels per dimension).

`https://iceshot.readthedocs.io/`

SM Video 1 (Incompressibility) *Toy simulation of the gradient descent dynamics* (3) *in a four-cell system. The negative gradient of the cost function (called incompressibility force) is displayed in red.*

SM Video 2 (3D hourglass) *Falling soft spheres in a hourglass domain with a spherical obstacle. Particles are progressively added in the funnel. The color of a particle only indicates the time when it has been added. See also Fig. 3A.*

SM Video 3 (3D tissue growth) *Growing cell aggregate in 3D following a basic somatic cell division process. See also Fig. 3B.*

SM Video 4 (Benchmark: deformable ellipsoids) *Benchmark experiment introduced in the main text for a system of* $N = 1000$ *self-propelled deformable ellipsoids with a discretization grid of size* $M = 512^3$. *See also Fig. 3C.*

SM Video 5 (Rod-shape particles) *Related to Fig. 2B: simulation of a system of rod-like particles with directional changes induced by deformation and bending effects. Long-range order spontaneously emerges after some time.*

SM Video 6 (Active Brownian Particles (1/3)) *Related to Fig. 4B: simulation of a system of active Brownian particles with deformability parameter* $\alpha = 0.5$. *The mean-square displacement of the particles is smaller but of the same order of magnitude than the theoretical value for independent point particles.*

SM Video 7 (Active Brownian Particles (2/3)) *Related to Fig. 4B: simulation of a system of active Brownian particles with deformability parameter* $\alpha = 2$. *As the deformability decreases, the mean-square displacement is drastically reduced.*

SM Video 8 (Active Brownian Particles (3/3)) *elated to Fig. 4B: simulation of a system of active Brownian particles with deformability parameter* $\alpha = 10$. *The particles behave as hard-spheres and self-organize into an optimal sphere packing configuration with almost no movement.*

SM Video 9 (Chemotaxis motion (1/2)) *Related to Fig. 2C: simulation of a system of 10 particles where the movement is induced by the shape deformation in response to a chemo-attractant gradient. With a particular choice of cost perturbation, particle migrate by adopting elongated shapes in the direction of increasing chemo-attractant gradient.*

SM Video 10 (Chemotaxis motion (2/2)) *Related to Fig. 2C: simulation of a system of 10 particles where the movement is induced by the shape deformation in response to a chemo-attractant gradient. With a particular choice of cost perturbation, particle migrate by adopting fan-like shapes orthogonal to the direction of increasing chemo-attractant gradient.*

SM Video 11 (3D Cell sorting (1/3)) *Related to Fig. 5A: simulation of a homogeneously mixed two-population cell aggregate with* $N = 128$ *and* $\overline{\eta} = 3$, $\overline{\gamma} = 1$ *and* $\overline{k} = 1$ *leading to the separation of the two populations.*

SM Video 12 (3D Cell sorting (2/3)) *Related to Fig. 5B: simulation of a homogeneously mixed two-population cell aggregate with* $N = 128$ *and* $\overline{\eta} = 0.3$, $\overline{\gamma} = 1$ *and* $\overline{k} = 1$ *leading to a checkerboard pattern state.*

SM Video 13 (3D Cell sorting (3/3)) *Related to Fig. 5A: simulation of a homogeneously mixed two-population cell aggregate with* $N = 128$ *and* $\overline{\eta} = 3$, $\overline{\gamma} = 2$ *and* $\overline{\eta\gamma k} = 0.8$ *leading to the internalization of the hardest cells (region A).*

SM Video 14 (3D Engulfment) *Related to Fig. 5C: simulation of a homogeneously mixed two-population cell aggregate with* $N = 128$ *and* $\overline{\eta} = 3$, $\overline{\gamma} = 2$ *and* $\overline{\eta\gamma k} = 0.8$ *leading to the engulfment of the hardest cells (region A) even with an initially totally segregated state.*

SM Video 15 (Crowd motion (1/2)) *Related to Fig. S2: simulation of crowd motion towards a single exit point with deformability parameter* $\alpha = 0.5$. *All the particles manage to escape at the price of large deformations.*

SM Video 16 (Crowd motion (2/2)) *Related to Fig. S2: simulation of crowd motion towards a single exit point with deformability parameter* $\alpha = 8$. *The particles behave as hard-spheres and end up in a stable congested arch state.*

SM Video 17 (Falling spheres (1/6)) *Related to Fig. S3: falling spheres with the deformation parameter* $\alpha = 0.75$. *Initially round shape cells adopt a columnar shape due to the external force and a high tolerance to deformation.*

SM Video 18 (Falling spheres (2/6)) *Related to Fig. S3: falling spheres with the deformation parameter* $\alpha = 2$. *The initially round particles are not so prone to deformation: they thus keep a convex shape and organize into a Voronoi-like configuration.*

SM Video 19 (Falling spheres (3/6)) *Related to Fig. S3: falling spheres with the deformation parameter* $\alpha = 10$. *The particles behave as hard-spheres.*

SM Video 20 (Falling spheres (4/6)) *Related to Fig. S4: falling spheres where the magnitude of the incompressibility force of the orange and blue spheres are respectively* $\tau_o = 8$ *and* $\tau_b = 3$.

SM Video 21 (Falling spheres (5/6)) *Related to Fig. S4: falling spheres where the magnitude of the incompressibility force of the orange and blue spheres are respectively* $\tau_o = 3$ *and* $\tau_b = 3$.

SM Video 22 (Falling spheres (6/6)) *Related to Fig. S4: falling spheres where the magnitude of the incompressibility force of the orange and blue spheres are respectively* $\tau_o = 1$ *and* $\tau_b = 3$.

SM Video 23 (Bubbles) *Related to Fig. S5: Simple simulation of a system of bubbles with random creation, explosion and fusion events at the interface between a fluid and the air.*

REFERENCES

1. D. S. Goodsell, Inside a living cell. *Trends Biochem. Sci.* **16**, 203–206 (1991).

2. B. Maury, A. Roudneff-Chupin, F. Santambrogio, J. Venel, Handling congestion in crowd motion modeling. *Netw. Heterog. Media* **6**, 485–519 (2011).

3. P. B. Armstrong, Cell sorting out: The self-assembly of tissues *In Vitro*. *Crit. Rev. Biochem. Mol. Biol.* **24**, 119–149 (1989).

4. R. Farhadifar, J.-C. Röper, B. Aigouy, S. Eaton, F. Jülicher, The influence of cell mechanics, cell-cell interactions, and proliferation on epithelial packing. *Curr. Biol.* **17**, 2095–2104 (2007).

5. E. Méhes, T. Vicsek, Collective motion of cells: From experiments to models. *Integr. Biol.* **6**, 831–854 (2014).

6. T. Ichikawa, H. T. Zhang, L. Panavaite, A. Erzberger, D. Fabrèges, R. Snajder, A. Wolny, E. Korotkevich, N. Tsuchida-Straeten, L. Hufnagel, A. Kreshuk, T. Hiiragi, An ex vivo system to study cellular dynamics underlying mouse peri-implantation development. *Dev. Cell* **57**, 373–386.e9 (2022).

7. S. Ichbiah, F. Delbary, A. McDougall, R. Dumollard, H. Turlier, Embryo mechanics cartography: Inference of 3D force atlases from fluorescence microscopy. *Nat. Methods* **20**, 1989–1999 (2023).

8. B. González-Bermúdez, G. V. Guinea, G. R. Plaza, Advances in micropipette aspiration: Applications in cell biomechanics, models, and extended studies. *Biophys. J.* **116**, 587–594 (2019).

9. J. M. Osborne, A. G. Fletcher, J. M. Pitt-Francis, P. K. Maini, D. J. Gavaghan, Comparing individual-based approaches to modelling the self-organization of multicellular tissues. *PLOS Comput. Biol.* **13**, e1005387 (2017).

10. G. W. Brodland, Computational modeling of cell sorting, tissue engulfment, and related phenomena: A review. *Appl. Mech. Rev.* **57**, 47–76 (2004).

11. P. Van Liedekerke, M. M. Palm, N. Jagiella, D. Drasdo, Simulating tissue mechanics with agent-based models: Concepts, perspectives and some novel results. *Comput. Part. Mech.* **2**, 401–444 (2015).

12. R. Z. Mohammad, H. Murakawa, K. Svadlenka, H. Togashi, A numerical algorithm for modeling cellular rearrangements in tissue morphogenesis. *Commun. Biol.* **5**, 239 (2022).

13. S. Kachalo, H. Naveed, Y. Cao, J. Zhao, J. Liang, Mechanical model of geometric cell and topological algorithm for cell dynamics from single-cell to formation of monolayered tissues with pattern. *PLOS ONE* **10**, e0126484 (2015).

14. L.-P. Chaintron, A. Diez, Propagation of chaos: A review of models, methods and applications. I. Models and methods. *Kinet. Relat. Models* **15**, 895–1015 (2022).

15. M. Nonomura, Study on multicellular systems using a phase field model. *PLOS ONE* **7**, e33501 (2012).

16. M. Nagayama, H. Monobe, K. Sakakibara, K. I. Nakamura, Y. Kobayashi, H. Kitahata, On the reaction–Diffusion type modelling of the self-propelled object motion. *Sci. Rep.* **13**, 12633 (2023).

17. N. Saito, S. Ishihara, Cell deformability drives fluid-to-fluid phase transition in active cell monolayers. *Sci. Adv.* **10**, eadi8433 (2024).

18. T. Hiraiwa, K. Shitara, T. Ohta, Dynamics of a deformable self-propelled particle in three dimensions. *Soft Matter* **7**, 3083–3086 (2011).

19. T. Ohta, Dynamics of Deformable Active Particles. *J. Physical Soc. Japan* **86**, 072001 (2017).

20. S. Osher, R. Fedkiw, *Level Set Methods and Dynamic Implicit Surfaces*, no. 153 in Applied Mathematical Sciences (Springer, New York Berlin Heidelberg, 2003).

21. C. S. Peskin, The immersed boundary method. *Acta Numerica* **11**, 479–517 (2002).

22. L. Yang, J. C. Effler, B. L. Kutscher, S. E. Sullivan, D. N. Robinson, P. A. Iglesias, Modeling cellular deformations using the level set formalism. *BMC Syst. Biol.* **2**, 68 (2008).

23. J. A. Glazier, F. Graner, Simulation of the differential adhesion driven rearrangement of biological cells. *Phys. Rev. E* **47**, 2128–2154 (1993).

24. F. Graner, J. A. Glazier, Simulation of biological cell sorting using a two-dimensional extended potts model. *Phys. Rev. Lett.* **69**, 2013–2016 (1992).

25. H. Honda, Description of cellular patterns by dirichlet domains: The two-dimensional case. *J. Theoret. Biol.* **72**, 523–543 (1978).

26. D. Sulsky, S. Childress, J. Percus, A model of cell sorting. *J. Theoret. Biol.* **106**, 275–301 (1984).

27. S. Kaliman, C. Jayachandran, F. Rehfeldt, A.-S. Smith, Limits of applicability of the voronoi tessellation determined by centers of cell nuclei to epithelium morphology. *Front. Physiol.* **7**, 551 (2016).

28. S. Miyazaki, T. Otani, K. Sugihara, T. Fujimori, M. Furuse, T. Miura, Mechanism of interdigitation formation at apical boundary of MDCK cell. *iScience* **26**, 106594 (2023).

29. M. Bock, A. K. Tyagi, J.-U. Kreft, W. Alt, Generalized Voronoi tessellation as a model of two-dimensional cell tissue dynamics. *Bull. Math. Biol.* **72**, 1696–1731 (2010).

30. D. Bi, X. Yang, M. C. Marchetti, M. L. Manning, Motility-driven glass and jamming transitions in biological tissues. *Phys. Rev. X* **6**, 021011 (2016).

31. R. I. Saye, J. A. Sethian, The Voronoi implicit interface method for computing multiphase physics. *Proc. Natl. Acad. Sci. U.S.A.* **108**, 19498–19503 (2011).

32. H. Honda, M. Tanemura, T. Nagai, A three-dimensional vertex dynamics cell model of space-filling polyhedra simulating cell behavior in a cell aggregate. *J. Theor. Biol.* **226**, 439–453 (2004).

33. H. H. Chen, G. W. Brodland, Cell-level finite element studies of viscous cells in planar aggregates. *J. Biomech. Eng.* **122**, 394–401 (2000).

34. A. Torres-Sánchez, M. Kerr Winter, G. Salbreux, Interacting active surfaces: A model for three-dimensional cell aggregates. *PLOS Comput. Biol.* **18**, e1010762 (2022).

35. J. Zhao, Y. Cao, L. A. DiPietro, J. Liang, Dynamic cellular finite-element method for modelling large-scale cell migration and proliferation under the control of mechanical and biochemical Cues: A study of re-epithelialization. *J. R. Soc. Interface* **14**, 20160959 (2017).

36. S. Runser, R. Vetter, D. Iber, SimuCell3D: Three-dimensional simulation of tissue mechanics with cell polarization. *Nat. Comput. Sci.* **4**, 299–309 (2024).

37. R. Vetter, S. V. M. Runser, D. Iber, PolyHoop: Soft particle and tissue dynamics with topological transitions. *Comput. Phys. Commun.* **299**, 109128 (2024).

38. P. Madhikar, J. Åström, J. Westerholm, M. Karttunen, *CellSim3D*: GPU accelerated software for simulations of cellular growth and division in three dimensions. *Comput. Phys. Commun.* **232**, 206–213 (2018).

39. A. Diez, SiSyPHE: A Python package for the simulation of systems of interacting mean-field particles with high efficiency. *J. Open Source Softw.* **6**, 3653 (2021).

40. A. Ghaffarizadeh, R. Heiland, S. H. Friedman, S. M. Mumenthaler, P. Macklin, PhysiCell: An open source physics-based cell simulator for 3-D multicellular systems. *PLOS Comput. Biol.* **14**, e1005991 (2018).

41. X. Kuang, G. Guan, C. Tang, L. Zhang, MorphoSim: An efficient and scalable phase-field framework for accurately simulating multicellular morphologies. *npj Syst. Biol. Appl.* **9**, 6 (2023).

42. J. Starruß, W. De Back, L. Brusch, A. Deutsch, Morpheus: A user-friendly modeling environment for multiscale and multicellular systems biology. *Bioinformatics* **30**, 1331–1332 (2014).

43. M. H. Swat, G. L. Thomas, J. M. Belmonte, A. Shirinifard, D. Hmeljak, J. A. Glazier, Multi-scale modeling of tissues using CompuCell3D. *Methods Cell Biol.* **110**, 325–366 (2012).

44. T. J. Sego, T. Comlekoglu, S. M. Peirce, D. W. Desimone, J. A. Glazier, General, open-source vertex modeling in biological applications using tissue forge. *Sci. Rep.* **13**, 17886 (2023).

45. F. Cooper, R. Baker, M. Bernabeu, R. Bordas, L. Bowler, A. Bueno-Orovio, H. Byrne, V. Carapella, L. Cardone-Noott, J. Cooper, S. Dutta, B. Evans, A. Fletcher, J. Grogan, W. Guo, D. Harvey, M. Hendrix, D. Kay, J. Kursawe, P. Maini, B. McMillan, G. Mirams, J. Osborne, P. Pathmanathan, J. Pitt-Francis, M. Robinson, B. Rodriguez, R. Spiteri, D. Gavaghan, Chaste: Cancer, heart and soft tissue environment. *J. Open Source Softw.* **5**, 1848 (2020).

46. Y. Brenier, A combinatorial algorithm for the euler equations of incompressible flows. *Comput. Methods Appl. Mech. Eng.* **75**, 325–332 (1989).

47. T. O. Gallouët, Q. Mérigot, A Lagrangian Scheme à La Brenier for the incompressible Euler equations. *Found. Comput. Math.* **18**, 835–865 (2018).

48. B. Lévy, Partial optimal transport for a constant-volume lagrangian mesh with free boundaries. *J. Comput. Phys.* **451**, 110838 (2022).

49. H. Leclerc, Q. Mérigot, F. Santambrogio, F. Stra, Lagrangian discretization of crowd motion and linear diffusion. *SIAM J. Numer. Anal.* **58**, 2093–2118 (2020).

50. D. P. Bourne, P. J. J. Kok, S. M. Roper, W. D. T. Spanjer, Laguerre tessellations and polycrystalline microstructures: A fast algorithm for generating grains of given volumes. *Philos. Mag.* **100**, 2677–2707 (2020).

51. D. Bourne, M. Pearce, S. Roper, Geometric modelling of polycrystalline materials: Laguerre tessellations and periodic semi-discrete optimal transport. *Mech. Res. Commun.* **127**, 104023 (2023).

52. M. Buze, J. Feydy, S. M. Roper, K. Sedighiani, D. P. Bourne, Anisotropic power diagrams for polycrystal modelling: Efficient generation of curved grains via optimal transport. arXiv:2403.03571 [cond-mat.mtrl-sci] (2024).

53. F. De Goes, C. Wallez, J. Huang, D. Pavlov, M. Desbrun, Power particles: An incompressible fluid solver based on power diagrams. *ACM Trans. Graph.* **34**, 1–11 (2015).

54. Z. Qu, M. Li, Y. Yang, C. Jiang, F. De Goes, Power plastics: A hybrid Lagrangian/Eulerian solver for mesoscale inelastic flows. *ACM Trans. Graph.* **42**, 1–11 (2023).

55. O. Busaryev, T. K. Dey, H. Wang, Z. Ren, Animating bubble interactions in a liquid foam. *ACM Trans. Graph.* **31**, 1–8 (2012).

56. J. Feydy, *Geometric Data Analysis, beyond Convolutions*, Ph.D. thesis, Université Paris-Saclay (2020).

57. G. Peyré, M. Cuturi, Computational optimal transport: With applications to data science. *Found. Trends Mach. Learn.* **11**, 355–607 (2019).

58. F. Santambrogio, *Optimal Transport for Applied Mathematicians: Calculus of Variations, PDEs, and Modeling*, vol. 87 of *Progress in Nonlinear Differential Equations and Their Applications* (Springer International Publishing, 2015), doi:10.1007/978-3-319-20828-2.

59. J. Kitagawa, Q. Mérigot, B. Thibert, Convergence of a newton algorithm for semi-discrete optimal transport. *J. Eur. Math. Soc.* **21**, 2603–2651 (2019).

60. A. Alpers, A. Brieden, P. Gritzmann, A. Lyckegaard, H. F. Poulsen, Generalized balanced power diagrams for 3D representations of polycrystals. *Philos. Mag.* **95**, 1016–1028 (2015).

61. B. Schmitzer, Stabilized sparse scaling algorithms for entropy regularized transport problems. *SIAM J. Sci. Comput.* **41**, A1443–A1481 (2019).

62. Q. Mérigot, A Multiscale Approach to Optimal Transport, in *Computer Graphics Forum* (Wiley Online Library, 2011), vol. 30, pp. 1583–1592.

63. B. Charlier, J. Feydy, J. A. Glaunès, F.-D. Collin, G. Durif, Kernel operations on the gpu, with autodiff, without memory overflows. *J. Mach. Learn. Res.* **22**, 1–6 (2021).

64. S.-Q. Xin, B. Lévy, Z. Chen, L. Chu, Y. Yu, C. Tu, W. Wang, Centroidal power diagrams with capacity constraints: Computation, applications, and extension. *ACM Trans. Graph.* **35**, 1–12 (2016).

65. D. L. Barton, S. Henkes, C. J. Weijer, R. Sknepnek, Active vertex model for cell-resolution description of epithelial tissue mechanics. *PLOS Comput. Biol.* **13**, e1005569 (2017).

66. V. Leech, F. N. Kenny, S. Marcotti, T. J. Shaw, B. M. Stramer, A. Manhart, Derivation and simulation of a computational model of active cell populations: How overlap avoidance, deformability, cell-cell junctions and cytoskeletal forces affect alignment. *PLOS Comput. Biol.* **20**, e1011879 (2024).

67. G. De Philippis, A. Figalli, The Monge–Ampère equation and its link to optimal transportation. *Bull. Amer. Math. Soc.* **51**, 527–580 (2014).

68. Y. Brenier, G. Loeper, A geometric approximation to the euler equations: The Vlasov-Monge-Ampère system. *GAFA, Geom. funct. anal.* **14**, 1182–1218 (2004).

69. G. W. Brodland, The differential interfacial tension hypothesis (DITH): A comprehensive theory for the self-rearrangement of embryonic cells and tissues. *J. Biomech. Eng.* **124**, 188–197 (2002).

70. R. Belousov, S. Savino, P. Moghe, T. Hiiragi, L. Rondoni, A. Erzberger, When time matters: Poissonian cellular potts models reveal nonequilibrium kinetics of cell sorting. arXiv:2306.04443 [cond-mat.stat-mech] (2023).

71. G. W. Brodland, H. H. Chen, The mechanics of heterotypic cell aggregates: Insights from computer simulations. *J. Biomech. Eng.* **122**, 402–407 (2000).

72. J.-L. Maître, H. Turlier, R. Illukkumbura, B. Eismann, R. Niwayama, F. Nédélec, T. Hiiragi, Asymmetric division of contractile domains couples cell positioning and fate specification. *Nature* **536**, 344–348 (2016).

73. D. P. Bourne, M. Pearce, S. M. Roper, Inverting laguerre tessellations: Recovering tessellations from the volumes and centroids of their cells using optimal transport. arXiv:2406.00871 [math.OC] (2024).

74. A. Buttenschön, L. Edelstein-Keshet, Bridging from single to collective cell migration: A review of models and links to experiments. *PLOS Comput. Biol.* **16**, e1008411 (2020).

75. A. Natale, Gradient flows of interacting laguerre cells as discrete porous media flows. arXiv:2304.05069 [math.NA] (2023).

76. D. P. Bourne, B. Schmitzer, B. Wirth, Semi-discrete unbalanced optimal transport and quantization. arXiv:1808.01962 [math.OC] (2018).

77. W. Schroeder, S. Tsalikis, M. Halle, S. Frisken, A high-performance surfacenets discrete isocontouring algorithm. arXiv:2401.14906 [cs.GR] (2024).

78. W. Schroeder, K. Martin, B. Lorensen, *The Visualization Toolkit (4th Ed.)* (Kitware, 2006).

79. C. Sullivan, A. Kaszynski, PyVista: 3D plotting and mesh analysis through a streamlined interface for the Visualization Toolkit (VTK). *J. Open Source Softw.* **4**, 1450 (2019).

80. Y. Ishimoto, Y. Morishita, Bubbly vertex dynamics: A dynamical and geometrical model for epithelial tissues with curved cell shapes. *Phys. Rev. E* **90**, 052711 (2014).
